# Global patterns in endemicity and vulnerability of soil fungi

**DOI:** 10.1111/gcb.16398

**Published:** 2022-09-02

**Authors:** Leho Tedersoo, Vladimir Mikryukov, Alexander Zizka, Mohammad Bahram, Niloufar Hagh‐Doust, Sten Anslan, Oleh Prylutskyi, Manuel Delgado‐Baquerizo, Fernando T. Maestre, Jaan Pärn, Maarja Öpik, Mari Moora, Martin Zobel, Mikk Espenberg, Ülo Mander, Abdul Nasir Khalid, Adriana Corrales, Ahto Agan, Aída‐M. Vasco‐Palacios, Alessandro Saitta, Andrea C. Rinaldi, Annemieke Verbeken, Bobby P. Sulistyo, Boris Tamgnoue, Brendan Furneaux, Camila Duarte Ritter, Casper Nyamukondiwa, Cathy Sharp, César Marín, Daniyal Gohar, Darta Klavina, Dipon Sharmah, Dong Qin Dai, Eduardo Nouhra, Elisabeth Machteld Biersma, Elisabeth Rähn, Erin K. Cameron, Eske De Crop, Eveli Otsing, Evgeny A. Davydov, Felipe E. Albornoz, Francis Q. Brearley, Franz Buegger, Geoffrey Zahn, Gregory Bonito, Inga Hiiesalu, Isabel C. Barrio, Jacob Heilmann‐Clausen, Jelena Ankuda, John Y. Kupagme, Jose G. Maciá‐Vicente, Joseph Djeugap Fovo, József Geml, Juha M. Alatalo, Julieta Alvarez‐Manjarrez, Kadri Põldmaa, Kadri Runnel, Kalev Adamson, Kari Anne Bråthen, Karin Pritsch, Kassim I. Tchan, Kęstutis Armolaitis, Kevin D. Hyde, Kevin K. Newsham, Kristel Panksep, Adebola A. Lateef, Liis Tiirmann, Linda Hansson, Louis J. Lamit, Malka Saba, Maria Tuomi, Marieka Gryzenhout, Marijn Bauters, Meike Piepenbring, Nalin Wijayawardene, Nourou S. Yorou, Olavi Kurina, Peter E. Mortimer, Peter Meidl, Petr Kohout, Rolf Henrik Nilsson, Rasmus Puusepp, Rein Drenkhan, Roberto Garibay‐Orijel, Roberto Godoy, Saad Alkahtani, Saleh Rahimlou, Sergey V. Dudov, Sergei Põlme, Soumya Ghosh, Sunil Mundra, Talaat Ahmed, Tarquin Netherway, Terry W. Henkel, Tomas Roslin, Vincent Nteziryayo, Vladimir E. Fedosov, Vladimir G. Onipchenko, W. A. Erandi Yasanthika, Young Woon Lim, Nadejda A. Soudzilovskaia, Alexandre Antonelli, Urmas Kõljalg, Kessy Abarenkov

**Affiliations:** ^1^ Mycology and Microbiology Center University of Tartu Tartu Estonia; ^2^ Institute of Ecology and Earth Sciences University of Tartu Tartu Estonia; ^3^ Department of Biology Philipps‐University Marburg Germany; ^4^ Department of Ecology Swedish University of Agricultural Sciences Uppsala Sweden; ^5^ Department of Mycology and Plant Resistance, School of Biology V.N. Karazin Kharkiv National University Kharkiv Ukraine; ^6^ Laboratorio de Biodiversidad y Funcionamiento Ecosistémico, Instituto de Recursos Naturales y Agrobiología de Sevilla (IRNAS), CSIC, and Unidad Asociada CSIC‐UPO (BioFun) Universidad Pablo de Olavide Sevilla Spain; ^7^ Departamento de Ecología, Instituto Multidisciplinar para el Estudio del Medio ‘Ramón Margalef’ Universidad de Alicante Alicante Spain; ^8^ Institute of Botany University of the Punjab Lahore Pakistan; ^9^ Centro de Investigaciones en Microbiología y Biotecnología‐UR (CIMBIUR) Universidad del Rosario Bogotá Colombia; ^10^ Institute of Forestry and Engineering Estonian University of Life Sciences Tartu Estonia; ^11^ BioMicro, Escuela de Microbiología Universidad de Antioquia UdeA Medellin Antioquia Colombia; ^12^ Department of Agricultural, Food and Forest Sciences University of Palermo Palermo Italy; ^13^ Department of Biomedical Sciences University of Cagliari Cagliari Italy; ^14^ Department Biology Ghent University Ghent Belgium; ^15^ Department of Biomedicine Indonesia International Institute for Life Sciences Jakarta Indonesia; ^16^ Department of Crop Science University of Dschang Dschang Cameroon; ^17^ Department of Ecology and Genetics Uppsala University Uppsala Sweden; ^18^ Departamento de Zootecnia Universidade Federal do Paraná Curitiba Brazil; ^19^ Department of Biological Sciences and Biotechnology Botswana International University of Science and Technology Palapye Botswana; ^20^ Natural History Museum of Zimbabwe Bulawayo Zimbabwe; ^21^ Centro de Investigación e Innovación para el Cambio Climático (CiiCC) Universidad SantoTomás Santiago Chile; ^22^ Latvian State Forest Research Insitute Silava Salaspils Latvia; ^23^ Department of Botany, Jawaharlal Nehru Rajkeeya Mahavidyalaya Pondicherry University Port Blair India; ^24^ College of Biological Resource and Food Engineering Qujing Normal University Qujing China; ^25^ Instituto Multidisciplinario de Biología Vegetal (CONICET) Universidad Nacional de Córdoba Cordoba Argentina; ^26^ Natural History Museum of Denmark Copenhagen Denmark; ^27^ Department of Environmental Science Saint Mary's University Halifax Canada; ^28^ Altai State University Barnaul Russia; ^29^ CSIRO Land and Water Wembley Western Australia Australia; ^30^ Department of Natural Sciences Manchester Metropolitan University Manchester UK; ^31^ Helmholtz Zentrum München Neuherberg Germany; ^32^ Utah Valley University Orem Utah USA; ^33^ Plant, Soil and Microbial Sciences Michigan State University East Lansing Michigan USA; ^34^ Faculty of Natural and Environmental Sciences Agricultural University of Iceland Hvanneyri Iceland; ^35^ Center for Macroecology, Evolution and Climate University of Copenhagen Copenhagen Denmark; ^36^ Department of Silviculture and Ecology Institute of Forestry of Lithuanian Research Centre for Agriculture and Forestry (LAMMC) Girionys Lithuania; ^37^ Plant Ecology and Nature Conservation Wageningen University & Research Wageningen The Netherlands; ^38^ ELKH‐EKKE Lendület Environmental Microbiome Research Group Eszterházy Károly Catholic University Eger Hungary; ^39^ Environmental Science Center Qatar University Doha Qatar; ^40^ Biology Department Stanford University Stanford California USA; ^41^ Department of Arctic and Marine Biology The Arctic University of Norway Tromsø Norway; ^42^ Research Unit Tropical Mycology and Plants‐Soil Fungi Interactions University of Parakou Parakou Benin; ^43^ Center of Excellence in Fungal Research Mae Fah Luang University Chiang Rai Thailand; ^44^ NERC British Antarctic Survey Cambridge UK; ^45^ Chair of Hydrobiology and Fishery Estonian University of Life Sciences Tartu Estonia; ^46^ Department of Plant Biology University of Ilorin Ilorin Nigeria; ^47^ Gothenburg Centre for Sustainable Development Gothenburg Sweden; ^48^ Department of Biology Syracuse University Syracuse New York USA; ^49^ Department of Environmental and Forest Biology State University of New York College of Environmental Science and Forestry Syracuse New York USA; ^50^ Department of Plant Sciences Quaid‐i‐Azam University Islamabad Pakistan; ^51^ Department of Genetics University of the Free State Bloemfontein South Africa; ^52^ Department of Environment Ghent University Ghent Belgium; ^53^ Mycology Working Group Goethe University Frankfurt am Main Frankfurt am Main Germany; ^54^ Institute of Agricultural and Environmental Sciences Estonian University of Life Sciences Tartu Estonia; ^55^ Center For Mountain Futures, Kunming Institute of Botany Chinese Academy of Sciences Kunming China; ^56^ Institut für Biologie Freie Universität Berlin Berlin Germany; ^57^ Institute of Microbiology Czech Academy of Sciences Prague Czech Republic; ^58^ Gothenburg Global Biodiversity Centre University of Gothenburg Gothenburg Sweden; ^59^ Instituto de Biología Universidad Nacional Autónoma de México Ciudad de México Mexico; ^60^ Instituto Ciencias Ambientales y Evolutivas Universidad Austral de Chile Valdivia Chile; ^61^ College of Science King Saud University Riyadh Saudi Arabia; ^62^ Department of Ecology and Plant Geography Moscow Lomonosov State University Moscow Russia; ^63^ Department of Biology, College of Science United Arab Emirates University Abu Dhabi UAE; ^64^ Department of Biological Sciences California State Polytechnic University Arcata California USA; ^65^ Department of Food Science and Technology University of Burundi Bujumbura Burundi; ^66^ School of Biological Sciences and Institute of Microbiology Seoul National University Seoul South Korea; ^67^ Centre for Environmental Sciences Hasselt University Hasselt Belgium; ^68^ Royal Botanic Gardens Kew UK; ^69^ University of Tartu Natural History Museum Tartu Estonia

**Keywords:** biodiversity, biogeography, climate change, conservation priorities, global change vulnerability, global maps, mycorrhizal fungi, pathogens, saprotrophs

## Abstract

Fungi are highly diverse organisms, which provide multiple ecosystem services. However, compared with charismatic animals and plants, the distribution patterns and conservation needs of fungi have been little explored. Here, we examined endemicity patterns, global change vulnerability and conservation priority areas for functional groups of soil fungi based on six global surveys using a high‐resolution, long‐read metabarcoding approach. We found that the endemicity of all fungi and most functional groups peaks in tropical habitats, including Amazonia, Yucatan, West‐Central Africa, Sri Lanka, and New Caledonia, with a negligible island effect compared with plants and animals. We also found that fungi are predominantly vulnerable to drought, heat and land‐cover change, particularly in dry tropical regions with high human population density. Fungal conservation areas of highest priority include herbaceous wetlands, tropical forests, and woodlands. We stress that more attention should be focused on the conservation of fungi, especially root symbiotic arbuscular mycorrhizal and ectomycorrhizal fungi in tropical regions as well as unicellular early‐diverging groups and macrofungi in general. Given the low overlap between the endemicity of fungi and macroorganisms, but high conservation needs in both groups, detailed analyses on distribution and conservation requirements are warranted for other microorganisms and soil organisms.

## Graphical abstract

Using a long‐read metabarcoding approach, we found that the endemicity of all fungi and most functional groups peaks in tropical habitats, including Amazonia, Yucatan and West‐Central Africa, with a negligible island effect compared with plants and animals. Fungi are predominantly vulnerable to drought, heat and land cover change, particularly in dry tropical regions with high human population density. Fungal conservation areas of highest priority include herbaceous wetlands, tropical forests and woodlands.

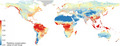

## 1 INTRODUCTION

Human activities affect nearly all habitats through changes in climate and land‐use, impacting vegetation cover and composition. These changes negatively influence many plant and animal species that have narrow environmental tolerances and limited dispersal capacities across anthropogenic landscapes (Schulte to Bühne et al., 2021). Anthropogenic impacts most strongly affect endemic species—that is, taxa with small distribution ranges and typically narrow ecological niches (Brook et al., 2008). The diversity of endemic plants and animals is higher in areas characterized by long‐term climatic stability, high precipitation, environmental heterogeneity and insularity. Unfortunately, these areas—which are mostly located in the tropics and subtropics—usually coincide with major human degradations of the environment (Kier et al., 2009; Sandel et al., 2020; Stein et al., 2014). Unlike the wealth of information that has accumulated for plants and animals, global patterns of fungal endemism and vulnerability to environmental change remain virtually unknown (Cameron et al., 2019; Guerra, Bardgett, et al., 2021; but see Davison et al., 2015). Only a few *Alnus*‐associated ectomycorrhizal (EcM) fungi (Põlme et al., 2013) and fewer than 100 soil‐borne saprotrophic and pathogenic fungi (Egidi et al., 2019) can be considered to be cosmopolitan, suggesting that the vast majority of fungal species may be endemic at least at a subcontinental scale (Talbot et al., 2014). This paucity of knowledge about the endemicity and vulnerability of fungi to global change is alarming, given the fundamental roles of fungi in soil carbon and nutrient cycling processes and as devastating crop and forest pathogens (Crowther et al., 2019; Wardle & Lindahl, 2014).

Comparative studies indicate that aboveground and belowground biodiversity is driven by different environmental predictors at local and global scales (Cameron et al., 2019; Le Provost et al., 2021). This suggests differential responses of macro‐ and microorganisms to land use and climate change (Guerra, Delgado‐Baquerizo, et al., 2021). Despite the vast global climatic gradients, soil pH appears to be the main driver of microbial diversity. For example, fungal richness peaks at weakly acidic soils (Tedersoo, Bahram, Põlme, et al., 2014; Tedersoo et al., 2022), whereas bacteria and protists are highest in neutral soils (Aslani et al., 2022; Bahram et al., 2018; Delgado‐Baquerizo, Oliverio, et al., 2018). Another study that did not measure pH found that the fungal diversity peaked at lower temperatures, but dominant fungal species were most‐influenced by mean annual temperature (Větrovský et al., 2019). As for plants and animals (Foden et al., 2019; Pacifici et al., 2015), soil fungal communities are likely vulnerable to global change drivers. For instance, high temperature stress (Barcenas‐Moreno et al., 2009; Malcolm et al., 2008; Misiak et al., 2021) and prolonged drought (de Vries et al., 2018; Schmidt et al., 2018) can alter fungal growth, functionality and community composition. Likewise, changes in land use that result in habitat fragmentation may lead to shifts in the prevalence of pathogenic, mutualistic and free‐living fungal groups (Brinkmann et al., 2019; Le Provost et al., 2021; Makiola et al., 2019; Rodriguez‐Ramos et al., 2021).

Compared with macroorganisms, information about the conservation needs of fungi and other microbes is very limited. While thousands of plant and animal species are listed as threatened on the International Union for Conservation of Nature (IUCN) global Red List, only 262 out of an estimated 2.2–3.8 million fungal species (Hawksworth & Lücking, 2017) have been listed as such. The majority of these are from high‐income countries in temperate regions (IUCN, 2021) and are from fungal groups that form conspicuous macroscopic fruiting bodies (Cao et al., 2021). However, the vast majority of fungi produce no, microscopic or inconspicuous fruiting bodies and are therefore difficult to survey using traditional morphology‐ and culturing‐based identification methods, which have hampered assessments of their distribution (Gonçalves et al., 2021). Consequently, we have no information for which parts of the world fungal conservation needs are the highest.

Here we used the most advanced high‐resolution sequencing technology to globally survey soil fungi and assess their endemicity and vulnerability to global change. We hypothesized that (i) as observed for plants and animals (Rosauer & Jetz, 2015), the endemicity of fungi is relatively higher in the tropics due to greater regional‐scale climatic stability and also coincides with that of plants and animals and (ii) the vulnerability of fungi to global change is highest in habitats experiencing the strongest warming effects (e.g., polar regions) and intensive land use (e.g., dry tropics). We predicted that endemicity and vulnerability patterns are more evident for macrofungi that require abundant resources for building reproductive structures and for obligately biotrophic groups compared with saprotrophic microfungal groups. From these findings, we identify regions where fungal conservation may be the most warranted, and propose global conservation priorities.

## 2 MATERIALS AND METHODS

### 2.1 Data sets

**FIGURE 1:**
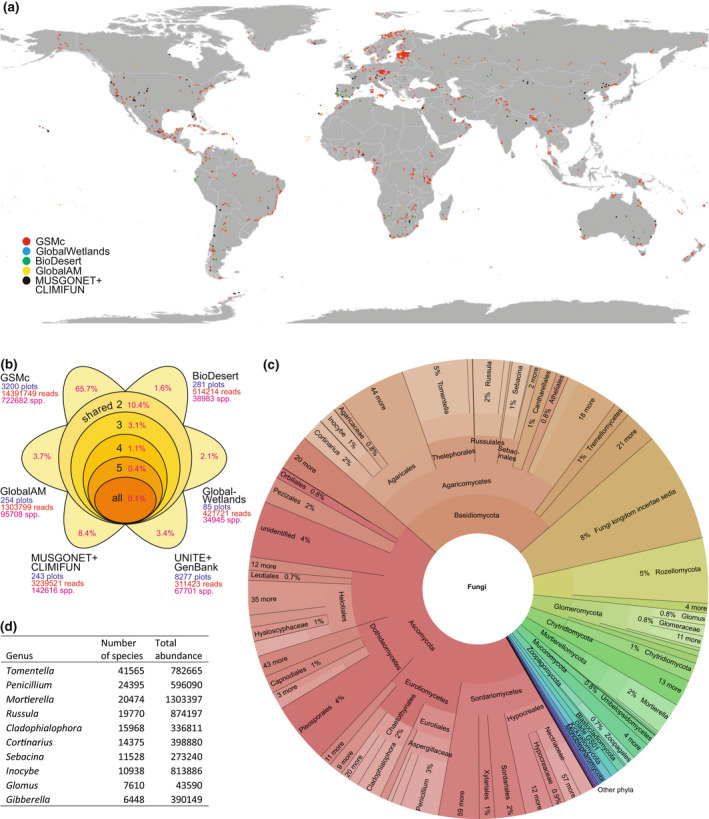
Distribution of samples and fungal species across data sets. (a) Global sampling map, with different colours representing different data sets; (b) species distribution of fungi among data sets, with the proportion of unique and shared species indicated; (c) Krona chart indicating the taxonomic distribution of fungal species (interactive chart can be browsed at https://plutof.ut.ee/#/doi/10.15156/BIO/2483900); and (d) species richness and total read abundance of the top 10 most diverse fungal genera.

To study fungal endemicity and vulnerability to global change, we combined data from the Global Soil Mycobiome consortium (GSMc) open data set (Tedersoo, Mikryukov, et al., 2021) with materials from five other global soil biological surveys (Figure 1)—BIODESERT (Maestre et al., 2022), MUSGONET (including the natural sites in Delgado‐Baquerizo et al., 2021), CLIMIFUN (Bastida et al., 2021), GlobalAM (Davison et al., 2021), GlobalWetlands (Bahram et al., 2022) as well as Sanger sequence data from soil‐inhabiting fungi obtained from the UNITE database (Nilsson et al., 2019) covering GenBank. We obtained soil DNA from these five surveys and performed new DNA metabarcoding analyses following the protocols outlined for the GSMc data set (Tedersoo, Mikryukov, et al., 2021).

All data sets included information on geographical coordinates and soil pH. Based on geographical coordinates, we assigned the following climatic and land‐cover metadata to the samples: (i) CHELSA v2.1 bioclimatic variables for the period 1981–2010 (Karger et al., 2017), (ii) CHELSA‐TraCE21k v1.0. for the last glacial maximum (LGM; Karger et al., 2021), and (iii) CHELSA v2.1 climate extrapolations for the year 2070 following the RCP8.5 global warming scenario with SSP5 socioeconomic conditions and the GFDL‐ESM4 global circulation model (Karger et al., 2020); (iv) normalized difference vegetation index (NDVI; Filipponi et al., 2018); (v) SoilGrids v.2 soil pH from 0 to 5 cm depth (Poggio et al., 2021); (vi) land‐cover type using Copernicus classification v.3 (Buchhorn et al., 2020) for the year 2015; and (vii) human footprint index based on the Land‐Use Harmonization (LUH2; Hurtt et al., 2020) for the year 2015 extrapolation. Based on original descriptions of vegetation (age, cover, relative abundance of species, fire history) or remote sensing data (Google Earth Pro; https://earth.google.com/), samples were assigned to biomes (Olson et al., 2001) and land‐cover types. Based on *z*‐transformed differences in all 19 present and LGM bioclimatic variables, we calculated for each sample an averaged LGM climate change index. Furthermore, for each sample we estimated the human footprint index as the cumulative sum of land‐use state transitions, with the year 1960 used as a baseline.

### 2.2 Molecular analysis and functional assignments

To infer fungal species and taxonomy, we used a long‐read sequencing approach involving the ribosomal RNA 18S gene V9 subregion, internal transcribed spacer 1 (ITS1), 5.8S gene and ITS2 to enhance taxonomic resolution and accuracy. We used degenerate, universal eukaryotic primers to cover as many divergent taxa within the fungi and micro‐eukaryotes as possible (Tedersoo, Albertsen, et al., 2021). The amplicon samples were prepared in 82 PacBio SMRTbell sequencing libraries and sequenced on 48 PacBio Sequel 8M SMRT cells. The obtained reads were quality‐filtered, demultiplexed to samples, trimmed to include only the full‐length ITS region, and clustered to operational taxonomic units (conditionally termed as species) at 98% sequence similarity, which roughly corresponds to species‐level divergence. Taxonomy was assigned based on information from the 10 best BLASTn matches against the UNITE 9.1 beta data set (https://doi.org/10.15156/BIO/1444285). The resulting species‐by‐sample matrices were manually checked library‐wise for external and cross‐contamination and rates of index switching artefacts. We excluded several samples for which we suspected contamination, and removed rare occurrences of dominant species using the following thresholds: abundances = 1 for species with total abundance of >99 and abundances = 2 for species with total abundance of >999.

Based on FungalTraits 1.3 (Põlme et al., 2020), species belonging to the kingdom Fungi were assigned to functional groups based on ecological or physiological characters: (i) arbuscular mycorrhizal (AM) fungi (including all Glomeromycota but excluding all Endogonomycetes, owing to the paucity of information distinguishing AM species from free‐living species); (ii) EcM fungi (excluding dubious lineages); (iii) non‐EcM Agaricomycetes (mostly saprotrophic fungi with usually macroscopic fruiting bodies); (iv) molds (including Mortierellales, Mucorales, Umbelopsidales and Aspergillaceae and Trichocomaceae of Eurotiales and *Trichoderma* of Hypocreales); (v) putative pathogens (including plant, animal and fungal pathogens as primary or secondary lifestyles); (vi) opportunistic human parasites (OHPs; excluding Mortierellales); (vii) yeasts (excluding dimorphic yeasts); and (viii) other unicellular (non‐yeast) fungi (including chytrids, aphids, rozellids, and other early‐diverging fungal lineages). Other groups such as lichen‐forming fungi were not considered, owing to their relative infrequency in the soil samples and across ecoregions. Among these groups, mostly non‐EcM Agaricomycetes and EcM fungi include many red‐listed species of macrofungi, with fruiting bodies that are conch‐shaped (polypores), resupinate (corticioids), or stipitate (agarics, boletes, etc.) and are hence considered to be of higher conservation interest because of their charismatic appearance (Cao et al., 2021; IUCN, 2021). Here, we test whether this approach is also justified from the endemicity and vulnerability perspectives.

### 2.3 Endemicity

**FIGURE 2:**
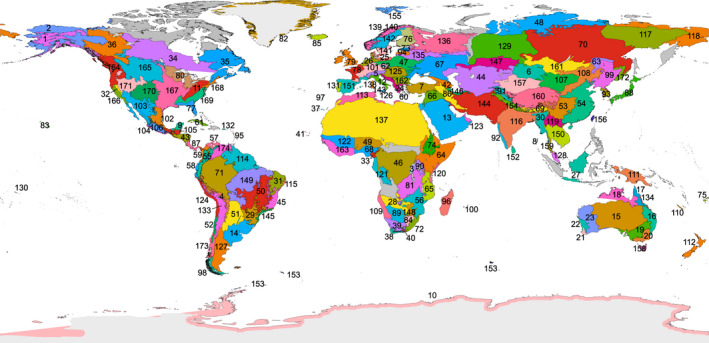
Distribution of 174 ecoregions used in endemicity analyses. Ecoregions excluded from the analyses due to the lack of data are indicated in grey. Their definition and relationship with Olson's ecological regions are given in Table S1.

To infer endemicity patterns in fungi, samples were assigned to ecological regions (Olson et al., 2001) based on their geographical coordinates, allowing a 10‐km buffer zone between terrestrial ecological regions and water (due to low resolution of the map layers in shore areas). Based on climatic and floristic similarities and data availability (Kier et al., 2009), the ecological regions were further subjectively aggregated into larger areas or split into smaller, geographically distinct units, which we refer to as ecoregions (Figure 2; Table S1). Each of the 174 ecoregions covered 1–45 soil samples, with surplus samples excluded randomly from two intensively sampled ecoregions. The ecoregions were allowed to include unlimited Sanger‐sequenced sites from UNITE. We distinguished the ecoregions located on islands and determined their minimum distance to nearest continents or larger islands using the Google Earth Pro ground distance measurement tool.

Using the *betapart* package v.1.5.4 (Baselga & Orme, 2012) of R v.4.1.10 (R Core Team, 2021), five indices of endemism—the number of endemic species (weight = 16.7%), proportion of endemic species (weight = 16.7%), mean maximum geographical range of taxa (weight = 33.3%), Jaccard index (weight = 16.7%) and beta‐sim index (weight = 16.7%)—were calculated based on the community matrix (Crisp et al., 2001; Villéger & Brosse, 2012; Box S1). The first two and the last two indices reflect similar aspects of endemicity and were therefore downweighed when averaging these indices based on *z*‐score transformation. To account for differences in sampling intensity, we calculated residuals for the numbers of all species and endemic species by regressing these against the logarithmically transformed number of samples and sequencing depth (Table S2).

Of the indices used, only the number of endemic taxa and proportion of endemic taxa were significantly positively correlated with fungal species richness (*r* = .707 and *r* = .212, respectively). Furthermore, species richness was not included among the best predictors of averaged endemicity, indicating that these metrics are independent. Endemicity indices of all fungi and functional groups were subjected to random forest machine learning analysis to pre‐select the 10 most important environmental variables for general linear modelling (GLM). We used the variance (as coefficient of variation) and averaged values of bioclimatic variables, area, latitude, longitude, altitude, and soil pH as well as continents (dummy variables) to explain endemicity. GLMs were fitted using second‐order polynomial terms for continuous variables. Only significant variables (*p* < .050; *r* ^2^ > .020) were kept in the final models. Based on the predictions revealed by GLMs, endemicity maps were constructed using the *sf* v.1.0.5 package (Pebesma, 2018) of R.

### 2.4 Global change vulnerability

Fungal vulnerability was estimated based on high‐throughput sequencing data at the soil sample level. The vulnerability of soil fungal groups was estimated relative to three global change drivers, viz. heat (maximum monthly temperature), drought (negative of inverse hyperbolic sine‐transformed precipitation in the driest quarter) and land cover change, for the year 2070 (relative to a 2015 baseline) using the community‐mean percentile vulnerability index (*V*_2_; Smith, Jovan, et al., 2020). This index is based on averaging percentiles of all species at a given global change driver value.

$$V_{2i}=\left(\frac{\sum_{j=1}^{n}a_{\mathrm{ij}}F_{j}\left(x_{i}\right)}{\sum_{j=1}^{n}a_{\mathrm{ij}}}\right)\times 100$$

where $a_{\mathrm{ij}}$ is the presence (0 or 1) of species *j* at site *i*, *F*_*j*_(*x*_*i*_) the percentile of species *j* given site parameter value *x*_*i*_, *x*_*i*_ the parameter value of site *i* and *n* the total number of species observed. Precipitation in the driest quarter was selected as a proxy for drought because bioclimatic variables cover larger areas (including islands) and offer greater resolution compared with other measures of soil water content and indicators of drought. The vulnerability scores were calculated for each soil sample using the *vuln* v.0.0.05 (Smith, Jovan, et al., 2020) package of R. We also constructed the average vulnerability score by equally weighing all components based on *z*‐scores. The vulnerability scores were unrelated to sequencing depth and sample size.

We performed a similar random forest and GLM modelling exercise to determine the main predictors of vulnerability as described above but allowed two‐way interaction terms between categorical and continuous predictors and used a more relaxed threshold for retaining variables in the model (*p* < .001; *R* ^2^ > .01) due to greater sample size. To predict vulnerability scores for each global driver and estimate their prediction uncertainty, thin plate splines (basis dimensionality = 3) were fitted using a generalized additive model with the *mgcv* v.1.8–38 (Wood, 2011) package of R. To incorporate the spatial autocorrelation signal, we calculated residuals at the sampling sites and used inverse distance weighting to interpolate residuals beyond the sampling sites. To obtain final vulnerability predictions, interpolated residuals were added to the results based on the predicted regression part (Hengl & MacMillan, 2019). By using the relative vulnerability values, we also prepared the map of fungal vulnerability ascribed to each of the three components. Vulnerability maps were visualized using the raster v.3.5–9 (Hijmans et al., 2021) package of R.

The maps for conservation priorities were calculated for all fungi using the same sampling points used for the vulnerability analyses, except for points corresponding to cropland and urban and village land cover types. For each sampling point, the respective average endemicity, γ‐diversity and vulnerability scores were *z*‐transformed, followed by adding a constant (5, to exclude negative values), multiplied (to downweight areas with any low values), and used in a regression approach (Table S3) as described for vulnerability.

## 3 RESULTS AND DISCUSSION

### 3.1 General findings

We used the recently generated Global Soil Mycobiome consortium data set (GSMc; 3200 plots, Tedersoo, Mikryukov, et al., 2021) along with data from five other global soil surveys (Figure 1) and international nucleotide sequence databases to determine the diversity and endemicity of fungal functional groups—viz. AM fungi, ectomycorrhizal (EcM) fungi, non‐EcM Agaricomycetes (mostly saprotrophic macrofungi), molds, pathogens, OHPs (mostly thermophilic saprotrophs), early‐diverging unicellular lineages and yeasts. Compared with previous meta‐analytical approaches (e.g., Větrovský et al., 2019), our cumulative data comprise the largest available globally standardized database based on directly comparable soil sampling and long‐read molecular analysis protocols. Collectively, all data sets yielded 20,182,427 fungal reads composed of 905,841 “species”—operational taxonomic units (OTUs), each defined at <98% sequence similarity of the rRNA ITS barcode from all other OTUs. *Tomentella* (Basidiomycota), *Penicillium* (Ascomycota), and *Mortierella* (Mortierellomycota) were the most species‐rich genera recorded (Figure 1).

### 3.2 Fungal endemicity

**FIGURE 3:**
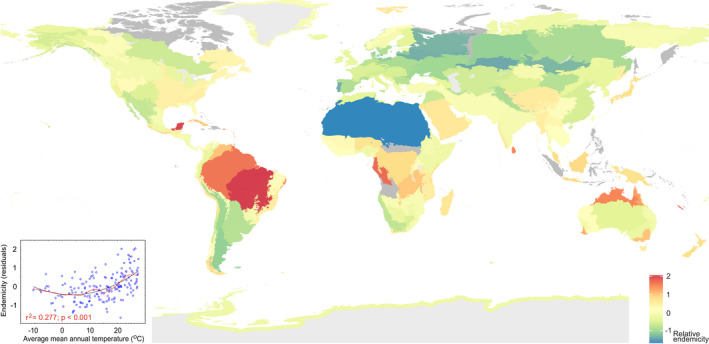
Average endemicity of soil fungi across ecoregions (defined in Figure 2). The inset graph indicates that endemicity has a positive and U‐shaped relationship with mean annual air temperature, the best predictor of endemicity. Grey ecoregions were excluded because of insufficient data.

To estimate relative endemism among the world's ecoregions (Figure 2, Table S4), we combined indices of community similarity, uniqueness, and species ranges into an overall endemicity index. We found that the endemicity of all fungi peaked in tropical rainforest and tropical woodland biomes of Amazonia (including cerrado), Yucatan, West‐Central Africa, Sri Lanka, and New Caledonia (Figure 3). Endemicity was positively related to mean annual air temperature (MAT; *R* ^2^_adj_ = .277; Figure 3) and soil acidity (*R* ^2^_adj_ = .108; Table S4).

While endemicity patterns of non‐EcM Agaricomycetes and AM fungi were similar to those shown for all fungi, different patterns were found for other functional groups (Figure S1). Endemicity of EcM fungi was related to high mean annual precipitation (*R* ^2^_adj_ = .147), with peaks in moist and dry tropical forests and temperate rainforests of Patagonia and New Zealand. Molds had relatively high endemicity in Amazonia, whereas pathogens and yeasts showed multiple endemicity hotspots. Molds (*R* ^2^_adj_ = .199) and pathogens (*R* ^2^_adj_ = .105) had relatively greater endemicity in strongly acidic or alkaline soils, indicating that extreme soil conditions may support unique soil biota, with limited effective dispersal across edaphically extreme habitats (Figure S1). Human footprint (see Section 2) had a weak negative effect on endemicity of all fungi (*R* ^2^_adj_ = .018), pathogens (*R* ^2^_adj_ = .015) and OHPs (*R* ^2^_adj_ = .056), suggesting that anthropogenic habitat loss or homogenization may affect endemic species (Finderup Nielsen et al., 2019). European ecoregions had the lowest endemicity for all fungi (*R* ^2^_adj_ = .065), pathogens (*R* ^2^_adj_ = .086) and unicellular fungi (*R* ^2^_adj_ = .035) compared with those of other subcontinents. Averaged current aerial bioclimatic variables better explained endemicity compared with the ranges of those variables or bioclimatic variables of soil and LGM. Climate change since the LGM had a weak positive effect on endemicity of molds (mean diurnal range and overall climate change: *R* ^2^_adj_ = .073) and OHPs (bioclimatic variables climate isothermality and mean diurnal range: *R* ^2^_adj_ = .056; Table S4).

**FIGURE 4:**
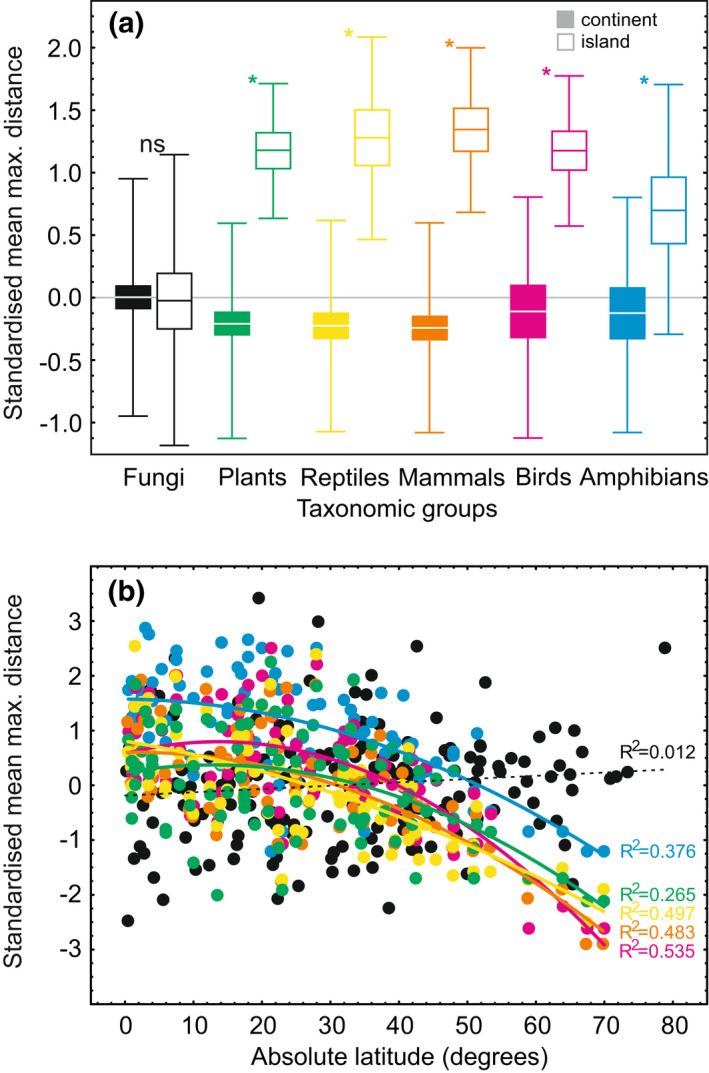
Endemicity patterns of fungi compared with plants and various vertebrate groups: (a) insularity effect and (b) latitudinal distribution. The analyses are based on world's ecoregions that differ for fungi compared with plants and animals (Kier et al., 2009). For all data sets, the mean maximum distance of all species was available and therefore taken as a measure of relative endemicity. For comparative purposes, these values were logarithm‐transformed, corrected for sequencing and sampling depth (only fungi) and *z*‐transformed. In (a) lines, boxes and error bars represent mean, SE and SD, respectively. In (b) the endemicity values were regressed against latitude using second‐order polynomial function; colours represent different organism groups as indicated in (a). For fungi, *n* = 156 and *n* = 28 for continent and island ecoregions, respectively; for other groups, *n* = 76 and *n* = 14 for continent and island ecoregions, respectively.

We found that patterns in fungal endemicity were relatively consistent among the five individual endemicity indices (Figure S1) and that, in agreement with our first hypothesis, they resembled endemicity patterns of vascular plants and animals, which exhibit major hotspots in wet tropical habitats (Barlow et al., 2018; Kier et al., 2009). However, in a striking contrast to plants and animals (Kier et al., 2009), fungal endemicity showed no detectable relationship with insularity (Figure 4). This lack of insularity effect may reflect the greater long‐distance dispersal capacity of fungal spores relative to plant propagules and animals (Golan & Pringle, 2017). The negligible effects of soil nutrients on fungal diversity (Tedersoo, Bahram, Põlme, et al., 2014; Tedersoo, Bahram, Ryberg, et al., 2014) and endemicity (this study) suggest that nutrient availability may not limit fungal growth or promote speciation.

The literature abounds with hypotheses, including narrower niche breadth, more asymmetric biotic interactions (i.e., greater specialization to symbiotic partners), long‐term climatic stability (LGM and earlier epochs), and more rapid evolution due to environmental energy (metabolic hypothesis; Brown, 2014; Vázquez & Stevens, 2004), to explain the greater macroorganism richness and endemicity found in the tropics than elsewhere. The observed negligible effects of the LGM suggest that climatic stability is not an important driver of fungal endemicity, a pattern which contrasts with that of plants and animals (Rosauer & Jetz, 2015). The greater phylogenetic diversity and mean neighbour taxonomic distance of fungi noted for the tropics (Tedersoo et al., 2018) reflect tropical origins for many lineages, as well as extensive radiation and rapid speciation of certain genera in higher latitude areas (Kennedy et al., 2012; Sánchez‐Ramírez et al., 2015; Tedersoo, Bahram, Ryberg, et al., 2014). On a global scale, plant diversity does not appear to be causally related to fungal diversity (Tedersoo, Bahram, Põlme, et al., 2014), but there is some evidence for stronger mutualistic plant–fungal interactions related to high rainfall (Põlme et al., 2018). Pathogenic interactions warrant further research in this respect, given their major importance as regulators of plant diversity (Chen et al., 2019). Tropical soil fungi have relatively greater dispersal limitations (Bahram et al., 2013) and narrower distribution ranges (this study), suggesting that high local diversity may contribute to greater regional‐scale endemicity.

### 3.3 Vulnerability to global change drivers

**FIGURE 5:**
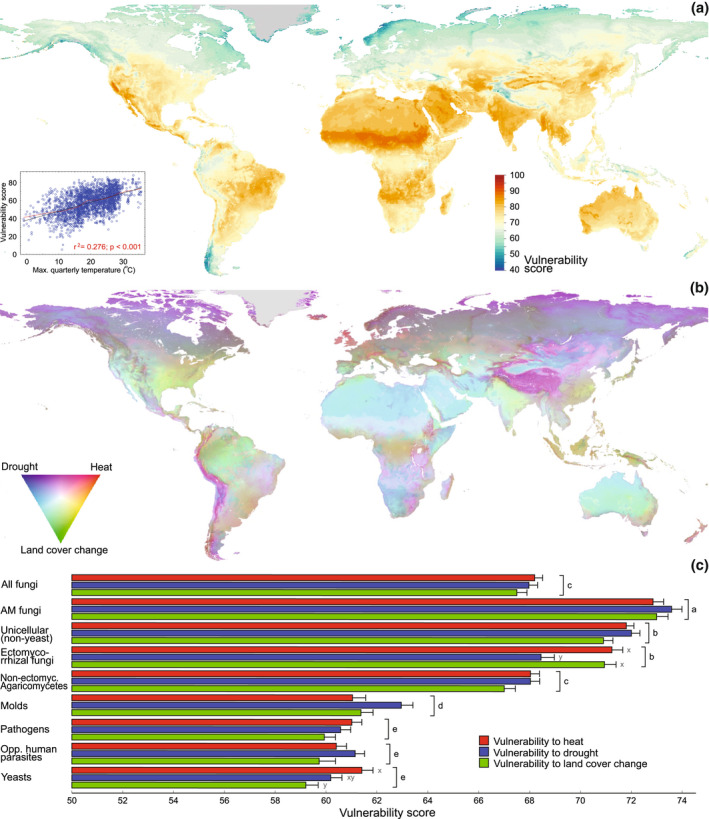
Vulnerability of fungi to global change drivers: (a) average vulnerability of all fungi to drought, heat and land cover change. The inset shows the near‐linear relationship of vulnerability to the air temperature of the warmest quarter. (b) Relative importance of predicted vulnerability of all fungi to drought, heat and land cover change as indicated by colour mixes. For example, the purple colour indicates high vulnerability to both heat and drought but low vulnerability to land cover change. (c) Relative importance of global change drivers for the predicted vulnerability of soil fungi and functional groups. Different letters indicate statistically significant (*p* < .001) differences among functional groups (a–e) and among global change drivers within functional groups (*x*–*z*). Mapping error estimates for panel (a) are given in Figure S4.

We evaluated the relative vulnerability of soil fungal functional groups by estimating the percentage of species occurring at their upper niche limits to three major global change drivers—land use (land cover change), heat (maximum monthly temperature), and drought (lowest quarterly precipitation). For all fungi taken together, predicted vulnerability to heat (best predictor: maximum monthly temperature; *R* ^2^_adj_ = .583) and drought (precipitation seasonality; *R* ^2^_adj_ = .456) were greatest in the drylands of tropical and subtropical latitudes. Vulnerability to land use change (best predictor: climate isothermality; *R* ^2^_adj_ = .145) peaked in the tropics (Figure S2; Table S5). The overall additive global change vulnerability was, thus, the highest in densely populated and drier tropical and subtropical regions, especially in India and the sub‐Saharan Sudanian savanna (Figure 5), and was positively associated with temperature‐related variables (cumulative *R* ^2^_adj_ = .382). Fungal functional groups had similar vulnerability patterns, which were mostly related to temperature (Figures S2 and S3; Table S5). Among fungal groups, average vulnerability scores were highest for AM and EcM symbionts and unicellular fungi, but these scores differed only slightly across the global change drivers (Figure 5c). The actual vulnerability was probably underestimated for biotrophic pathogens and EcM fungi because these groups associate with a limited number of plant species and are sometimes host‐specific (Kennedy et al., 2015). Therefore, the loss of one of only a few key symbiotic partners may greatly reduce the biotic niches of specialist fungi.

Patterns of vulnerability in fungi are somewhat similar to those of terrestrial plants and animals, and in agreement with our second hypothesis, vulnerability peaks in drylands prone to desertification (Warren et al., 2013), Arctic/alpine areas (cold‐adapted species) and regions with dense human populations (Watson et al., 2013). The relatively low vulnerability to heat in tundra‐inhabiting fungi can be explained by their relatively high‐temperature optima (Maynard et al., 2019; but see Misiak et al., 2021), acclimation (Romero‐Olivares et al., 2017) and poleward migration potentials, despite relatively greater predicted warming in Arctic ecosystems. Above certain tolerance thresholds, soil organisms may be physiologically constrained by increasing soil temperature and evaporation, lower soil water potentials and loss of oxygen due to greater respiration and faster decomposition, which result in hampered soil functioning and ecosystem multifunctionality (Delgado‐Baquerizo et al., 2017). Open areas are predicted to increase due to climate change and human activities. This will further expose soil to solar radiation and result in erosion and the loss of plant hosts for fungi. Losses of soil carbon and nutrients, but also anthropogenic nitrogen deposition, may exacerbate fungal vulnerability. These drivers are expected to be more influential locally (e.g., Correia et al., 2021; van der Linde et al., 2018) and are partly accounted for by land cover change in this study. Furthermore, while here we calculated average vulnerabilities by summing the effects of individual drivers, global change impacts tend to be synergistic (Rillig et al., 2019), so actual vulnerabilities may be much higher.

### 3.4 Implications for conservation

Most fungi and soil organisms do not enjoy the protection and conservation measures that are afforded to more charismatic animals and plants (Ducarme et al., 2013). Nonetheless, fungi and other soil biota are pivotal to soil and plant health, carbon and nutrient cycling, water storage, food security and many other ecosystem services. Their biodiversity should, hence, be brought to the focus of global sustainability thinking and conservation planning. For example, these organisms should be factored in when selecting protected areas, which are otherwise based on the distributions of plants or animals (Guerra, Bardgett, et al., 2021). The fact that many EcM and plant pathogenic fungal species are associated with specific host plants indicates that on the local scale it is not only the narrowly distributed species, but also those with unique or specific biotic associations, which require focused conservation measures. From the fungal perspective, it is particularly important to protect plant species that act as hubs in modules of biotic interaction networks, because these hub species typically associate with multiple, distinct fungal partners (Põlme et al., 2018). In other cases, certain unique plant species or higher taxonomic groups should be prioritized. For example, in southern South America, the drought‐sensitive tree family Nothofagaceae is the only group known to support EcM fungi that are mostly endemic to the region (Godoy & Marin, 2019).

Although the vulnerability scores to environmental change differed among fungal groups, their overall global patterns were similar. This suggests that broad habitat conservation measures may work for most fungal groups, including the macroscopic non‐EcM Agaricomycetes and EcM fungi as well as more cryptic pathogens and other groups. To accomplish this, fungi need to be incorporated into conservation frameworks (Gonçalves et al., 2021). Actions to fill existing information gaps at the local and global levels must also be taken, and global‐scale surveys should account for soil biodiversity assessments, complementing traditional collections‐based assessment with metabarcoding of environmental DNA. For fungi—and many other soil organisms—analyses of distribution and conservation requirements are now feasible based on large‐scale molecular surveys that can be highly standardized and rapidly performed. Such studies can distinguish cryptic species and ameliorate the enormous data coverage biases in GBIF and other global databases. Data produced by such studies can then be used in national conservation programs and global policy‐making initiatives, such as the System of Environmental Economic Accounting of the United Nations, World Biodiversity Forum and Post‐2020 Global Biodiversity Framework. Furthermore, promoting the red‐listing of endangered fungal species at the national and global levels is critical (FAO, 2020; IUCN, 2021). Fungi need active and specific inclusion in national and global conservation policies and strategies, not just passive and implicit protection.

**FIGURE 6:**
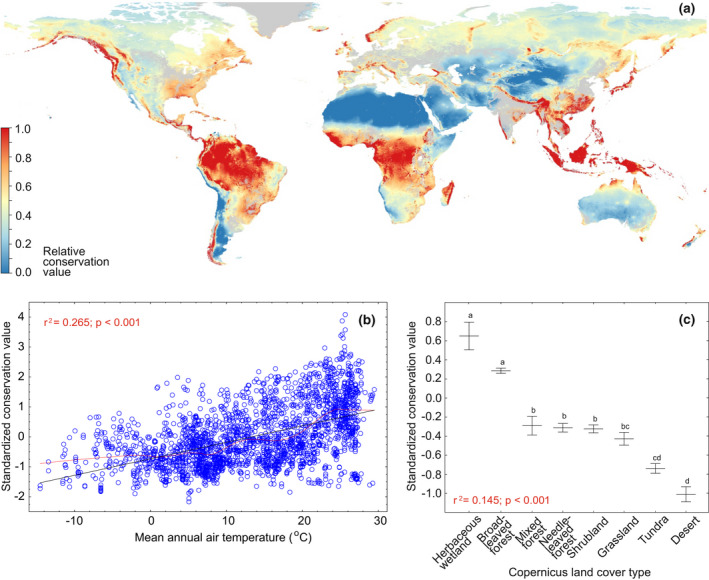
Conservation priority areas for all fungi (a) and their relationships with mean annual temperature (b) and Copernicus land cover types (c). In (b), black and red lines indicate best‐fitting linear and lowess functions, respectively. In (c), central lines and whiskers indicate mean and standard errors, respectively; letters above whiskers indicate statistically significant differences among land cover types.

Our study provides evidence that soil fungi may be highly vulnerable to global change, which needs to be considered when planning how to preserve these ecologically pivotal organisms in a changing world. As with plants and animals, fungi appear to be sensitive to the strong impacts of land cover change, low moisture and high temperatures on the taxonomic and functional composition of communities (Brinkmann et al., 2019; Makiola et al., 2019; this study). The endemicity of fungi is highest in tropical forest biomes, so conservation measures advocated for tropical plants and animals (Barlow et al., 2018; Brooks et al., 2006) are likely to conserve fungi. Tropical forests are under continued threat from deforestation and degradation driven by expanding agriculture, extractive industries and infrastructural projects (Bebbington et al., 2018). Conservation of herbaceous wetlands, tropical rainforests and tropical woodlands is supported by our global fungal conservation priority map that accounts for endemicity, vulnerability and γ‐diversity (Figure 6). Additionally, given the importance of soil pH for soil microbial diversity and composition (Aslani et al., 2022; Bahram et al., 2018; Delgado‐Baquerizo, Oliverio, et al., 2018; Tedersoo, Bahram, Põlme, et al., 2014; Tedersoo, Bahram, Ryberg, et al., 2014; Tedersoo et al., 2022), it is essential to prioritize areas with high pedodiversity or mixed landscapes including bogs, various forest types and grasslands. As a crucial measure, desertification and loss of soil organic matter need to be controlled by reducing the conversion of primary forest to crops and pasture (Smith, Calvin, et al., 2020). This is important, not only to prevent land degradation processes from impairing the diversity of fungi and other soil biota (Bach et al., 2020; Guerra, Bardgett, et al., 2021), but also to sustain the capacity of drylands to provide essential ecological functions and ecosystem services, such as soil fertility, carbon storage and food production for more than one billion people (Delgado‐Baquerizo, Eldridge, et al., 2018; Sivakumar, 2007).

## 4 CONCLUSIONS

Soil fungi show strong endemicity patterns, which differ between functional groups and are driven by both climatic and edaphic factors. Fungal groups also differ strongly in their relative vulnerability scores to global change, which peak in heavily populated dryland areas. Unfortunately, these are the very areas most prone to further land degradation and desertification, with the potential loss of many species. Fungal endemicity and vulnerability patterns only partly mirror those of vascular plants and animals, with virtually no insularity effects detected among fungi—a pattern that may be ascribed to their more efficient dispersal mechanisms. Global conservation efforts should include fungal biodiversity surveys alongside assessments of soil health, below‐ and aboveground feedbacks and areas of highest conservation priority, to secure the protection of keystone host species and natural habitats. Furthermore, they should include the monitoring of regional fungal communities over time, to detect relevant changes and provide early warning signals of impending change. The analysis for soil fungi presented here strongly suggests that microorganisms, and soil organisms in general, also deserve detailed assessments of their geographic distributions and conservation needs.

## AUTHOR CONTRIBUTIONS

Leho Tedersoo, Vladimir Mikryukov, Alexander Zizka, Mohammad Bahram, Nadejda A. Soudzilovskaia, Alexandre Antonelli, Urmas Kõljalg, and Kessy Abarenkov designed the study; Vladimir Mikryukov, Leho Tedersoo, Alexander Zizka, Marijn Bauters, Niloufar Hagh‐Doust, Sten Anslan and Oleh Prylutskyi analysed data; Leho Tedersoo, Manuel Delgado‐Baquerizo, Fernando T. Maestre, Jaan Pärn, Maarja Öpik, Mari Moora, Martin Zobel, Mikk Espenberg, and Ülo Mander contributed DNA extracts from global surveys; other authors contributed materials, data and/or chemical analyses; Leho Tedersoo wrote the first draft, and all authors contributed to the writing of the article.

## CONFLICT OF INTEREST

The authors declare no competing interests.

## Supporting information


Appendix S1

Figure S1

Table S1

Table S2

Table S3

## Data Availability

The data supporting the results is archived in Zenodo (https://doi.org/10.5281/zenodo.6983158 and https://doi.org/10.5281/zenodo.7027315) and PlutoF (https://doi.org/10.15156/BIO/2263453). The scripts for analyses are available via GitHub (https://github.com/Mycology‐Microbiology‐Center/Fungal_Endemicity_and_Vulnerability).
