# Supplementary material for: Global patterns in endemicity and vulnerability of soil fungi
Source: Glob Chang Biol. 2022 Sep 2;28(22):6696–710. doi: 10.1111/gcb.16398 (PMC9826061; doi:10.1111/gcb.16398)
Supplement: Supplementary file 1 — Appendix S1 [file GCB-28-6696-s001.pdf]

Calculation of endemicity indices

To estimate endemicity at the ecoregion level, we calculated five endemicity indices (E_1-E_5). Weighted endemicity indices were calculated based on these five indices using z-transformation of residuals in regression analyses against sequencing and sampling depth.

Five endemicity indices

E_1 Number of endemic species

$$E_1$$

E_2 Proportion of endemic species

$$E_2=\frac{E_1}{S}$$

S, total species richness in ecoregion

E_3 Mean maximum range of species (average weighted endemism; Crisp et al. 2001)

$$E_3=\frac{\sum_i^n d_{ri}}{S}$$

d_{ri}, range of species i

E_4 Jaccard index of similarity (Jaccard 1912)

$$E_4(C_1,C_2)=\frac{C_1 \cap C_2}{C_1 \cup C_2}$$

C, ecoregion communities

E_5 β_{SIM} index of similarity (multiple-site Simpson-based community similarity index, Baselga et al. 2010)

$$E_5=\frac{\sum_{i<j}\min(b_{ij},b_{ji})}{\sum_i(S_i-S_T)+\sum_{i<j}\min(b_{ij},b_{ji})}$$

S_i, number of species in site i

S_T, total number of species

bij and *bji*, number of species exclusive to sites i and j, respectively

Accounting for sampling and sequencing depth for endemicity indices

E_{nR} residuals of n endemicity index

$$E_{nR}=E_n-\hat{E}_n;$$
$$\hat{E}_n=\beta_1 Log(L_1+L_2)+\beta_2 Log(R_1+R_2)+ +\beta_3 Log(L_1)+\beta_4 Log(L_2)+\beta_5 Log(R_1)+ +\beta_6 Log(R_2)+\beta_7(L_1+L_2)+\beta_8(R_1+R_2)+ +\beta_9 L_1+\beta_{10}L_2+\beta_{11}R_1+\beta_{12}R_2+\varepsilon$$

L_1, number of soil sampling localities

L_2, number of UNITE localities

R_1, number of reads of soil samples

R_2, number of UNITE Sanger reads

Residuals are calculated based on the best-fitting model.

Standardizing residuals of endemicity indices

E_{nRz} Z-transformed E_{nR}

$$E_{nRz}=\frac{E_{nR}-\mu}{\sigma}$$

μ, mean E_{nR}

σ, standard deviation of E_{nR}.

Weighting standarded endemicity indices

E_{ave} weighted E_{nRz}

$$E_{ave}=\frac{E_{1Rz}+E_{2Rz}+2E_{3Rz}+E_{4Rz}+E_{5Rz}}{6}$$

E_3 receives double weight, because E_1 and E_2 as well as E_3 and E_4 are calculated on a similar basis.

Correlation matrix of endemicity indices

	E_{ave}	E_{1Rz}	E_{2Rz}	E_{3Rz}	E_{4Rz}	E_{5Rz}
E_{ave}	1.000	0.767	0.805	0.554	0.754	0.816
E_{1Rz}	0.767	1.000	0.806	0.188	0.452	0.540
E_{2Rz}	0.805	0.806	1.000	0.072	0.788	0.745
E_{3Rz}	0.554	0.188	0.072	1.000	0.169	0.270
E_{4Rz}	0.754	0.452	0.788	0.169	1.000	0.782
E_{5Rz}	0.816	0.540	0.745	0.270	0.782	1.000

Correlation matrix of average endemicity of fungal groups

	1	2	3	4	5	6	7	8	9
all fungi (1)	1.000	0.797	0.591	0.517	0.568	0.628	0.675	0.619	0.523
non-EcM Agaricomycetes (2)	0.797	1.000	0.790	0.398	0.461	0.470	0.460	0.595	0.532
AM fungi (3)	0.591	0.790	1.000	0.280	0.375	0.232	0.233	0.578	0.461
EcM fungi (4)	0.517	0.398	0.280	1.000	0.234	0.268	0.250	0.270	0.176
molds (5)	0.568	0.461	0.375	0.234	1.000	0.600	0.413	0.364	0.513
OHP (6)	0.628	0.470	0.232	0.268	0.600	1.000	0.809	0.159	0.354
pathogens (7)	0.675	0.460	0.233	0.250	0.413	0.809	1.000	0.264	0.331
unicellular fungi (8)	0.619	0.595	0.578	0.270	0.364	0.159	0.264	1.000	0.463
yeasts (9)	0.523	0.532	0.461	0.176	0.513	0.354	0.331	0.463	1.000

References

Baselga, A. (2010). Partitioning the turnover and nestedness components of beta diversity. *Global Ecology and Biogeography, 19,* 134-143.

Jaccard, P. (1912). The distribution of the flora in the alpine zone. *New Phytologist, 11*, 37–50.

BOX S1. Calculation of endemicity indices and correlation among standardised indices for all fungi and among functional groups.
