# Supplementary material for: Global patterns in endemicity and vulnerability of soil fungi
Source: Glob Chang Biol. 2022 Sep 2;28(22):6696–710. doi: 10.1111/gcb.16398 (PMC9826061; doi:10.1111/gcb.16398)
Supplement: Supplementary file 2 — Figure S1 [file GCB-28-6696-s002.pdf]

Non-mycorrhizal Agaricomycetes: average endemicity

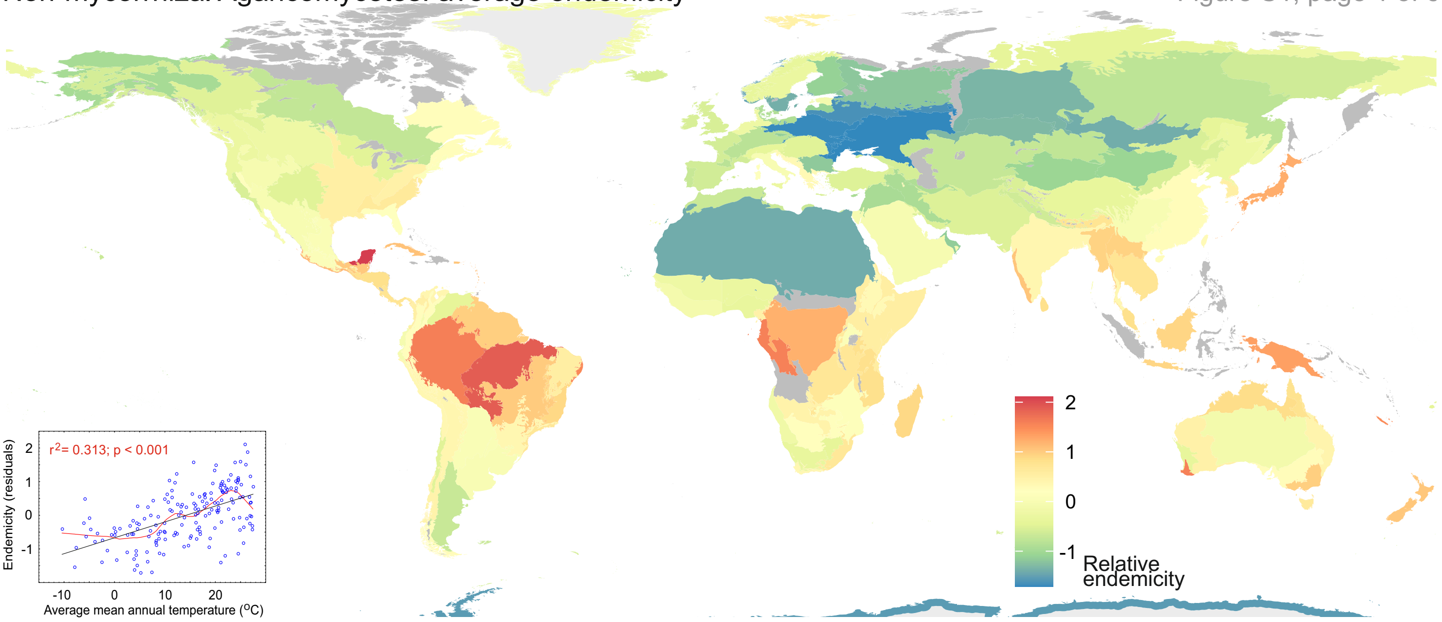

Arbuscular mycorrhizal fungi (only Glomeromycota): average endemicity

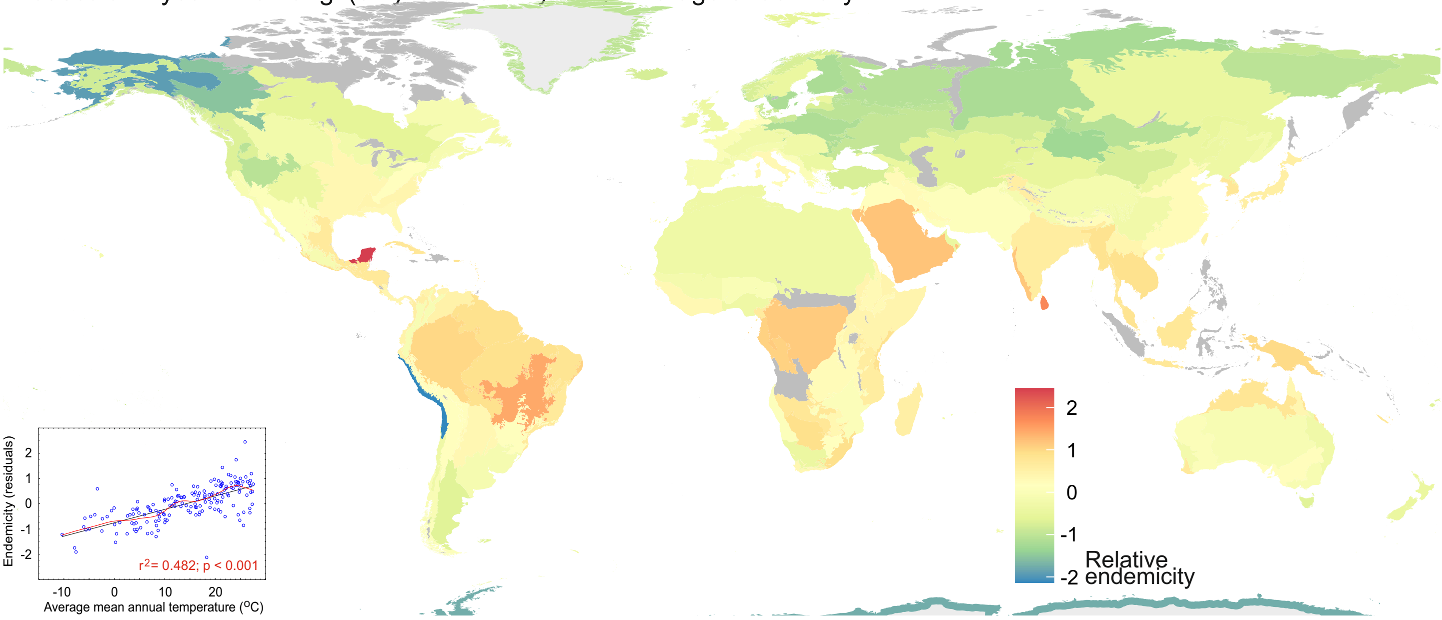

Ectomycorrhizal fungi: average endemicity

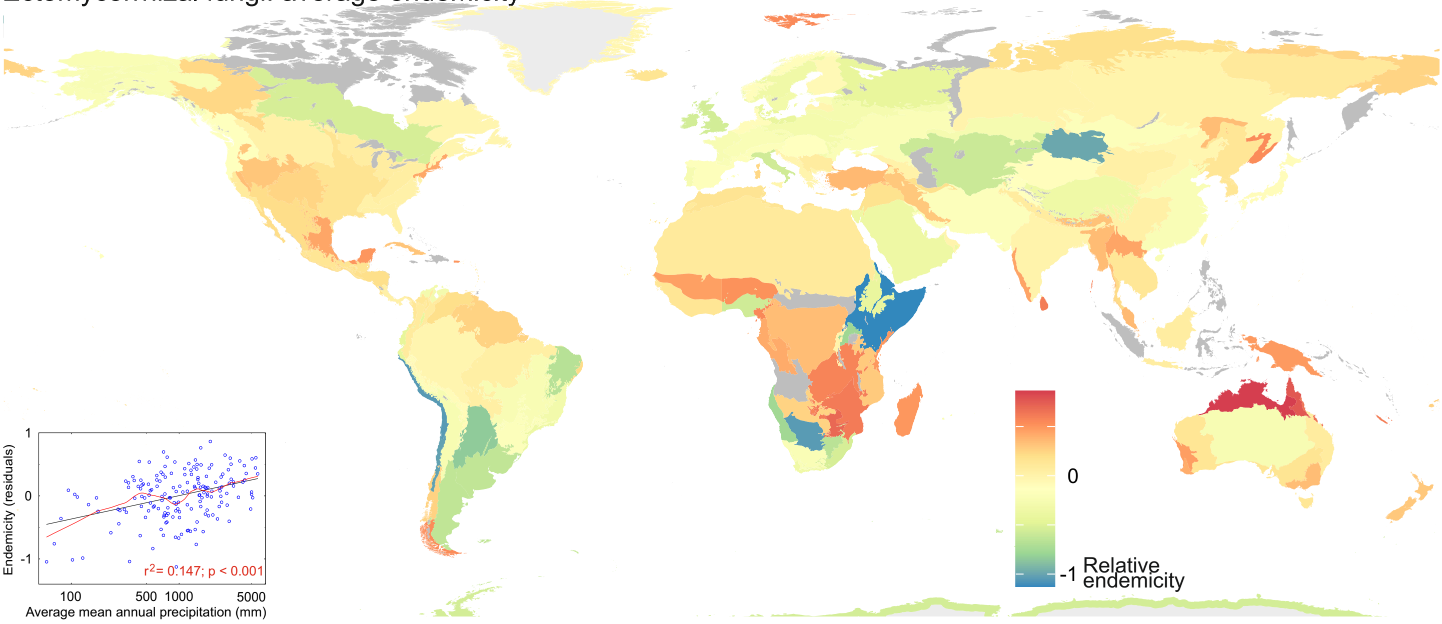

FIGURE S1 Average endemicity of fungal functional groups and five endemicity metrics of all fungi at the ecoregion scale. The insets indicate relationships to the best predictor (black line, best linear or quadratic fit; red line, lowess curve).

Molds: average endemicity

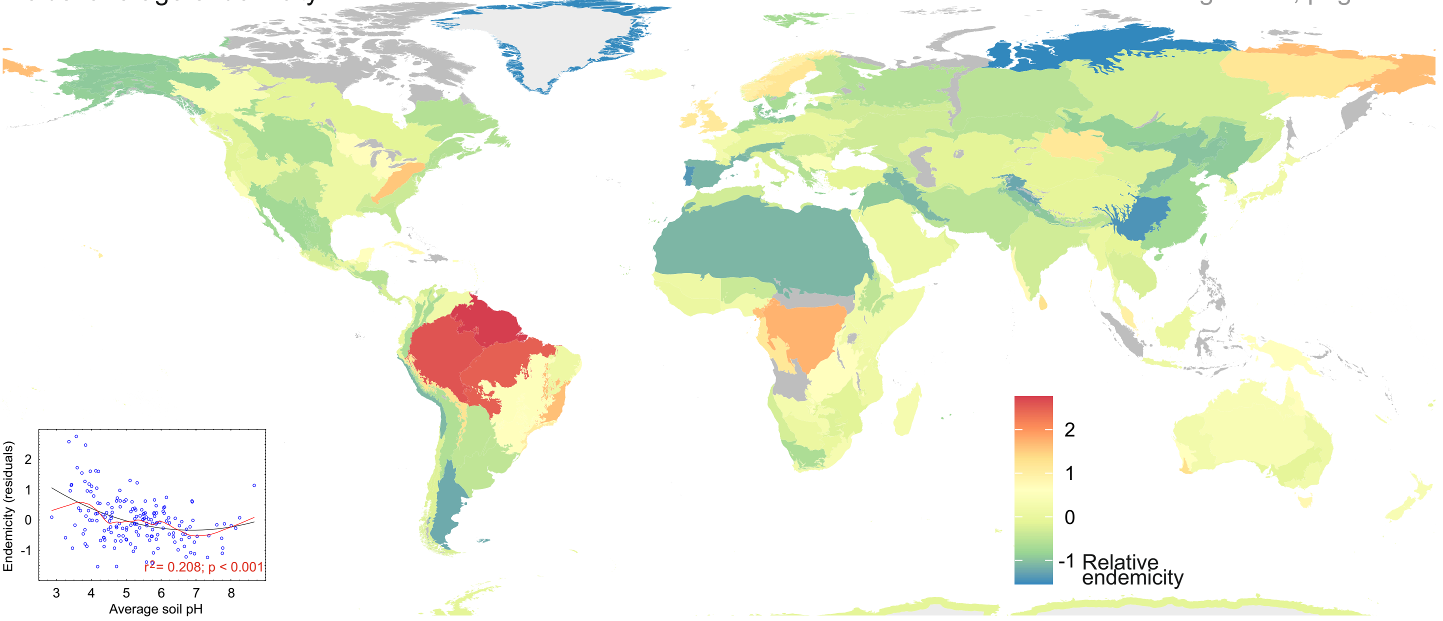

Opportunistic human parasites: average endemicity

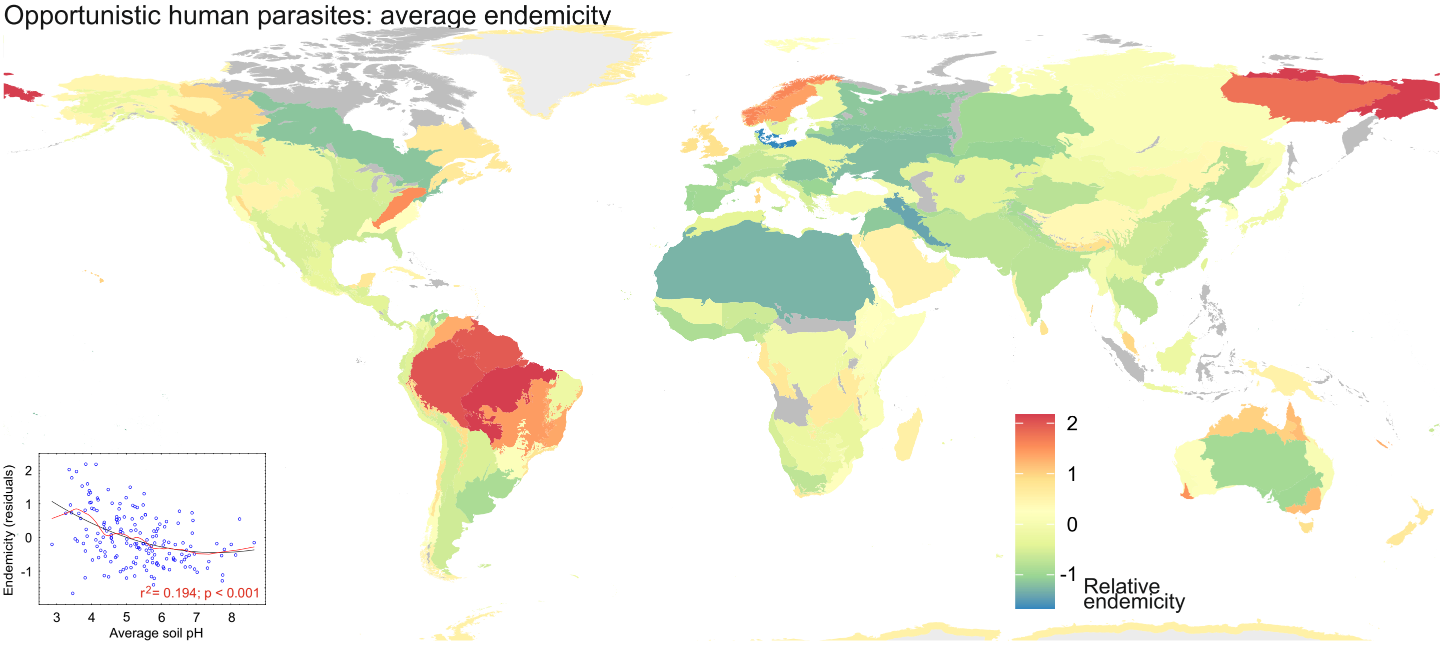

Pathogens: average endemicity

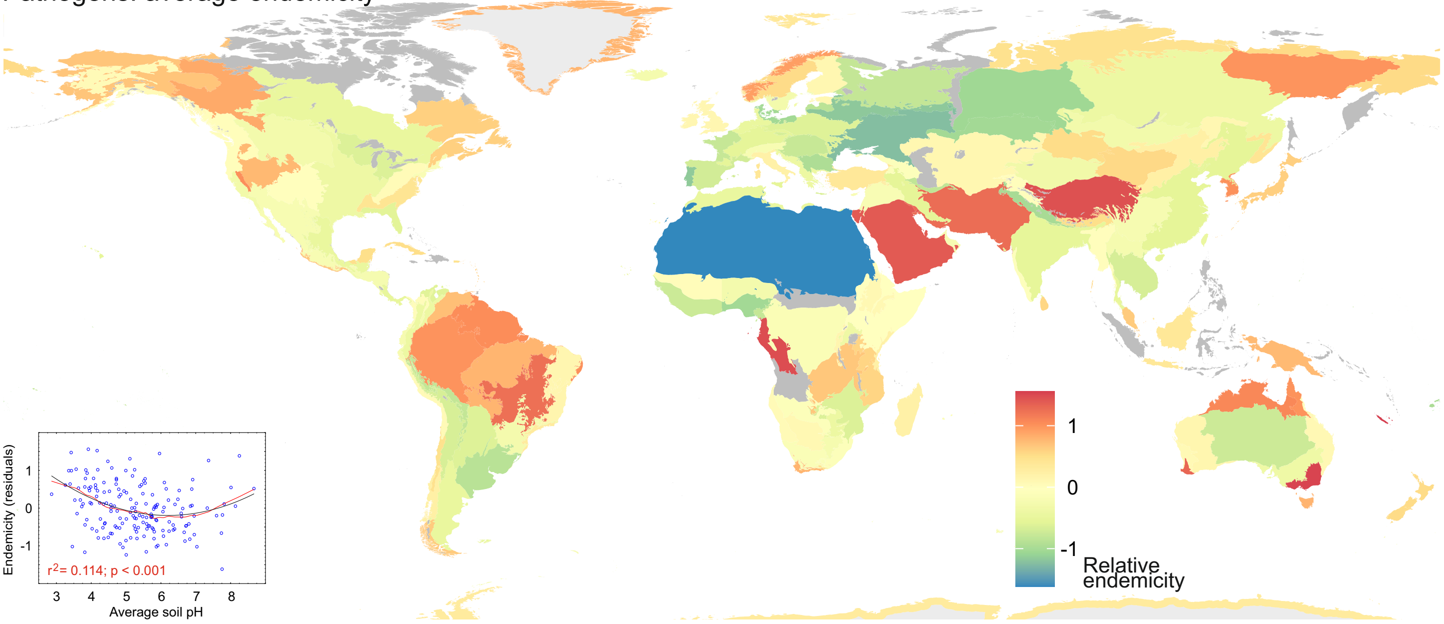

FIGURE S1 Average endemicity of fungal functional groups and five endemicity metrics of all fungi at the ecoregion scale. The insets indicate relationships to the best predictor (black line, best linear or quadratic fit; red line, lowess curve).

Unicellular fungi (non-yeast): average endemicity

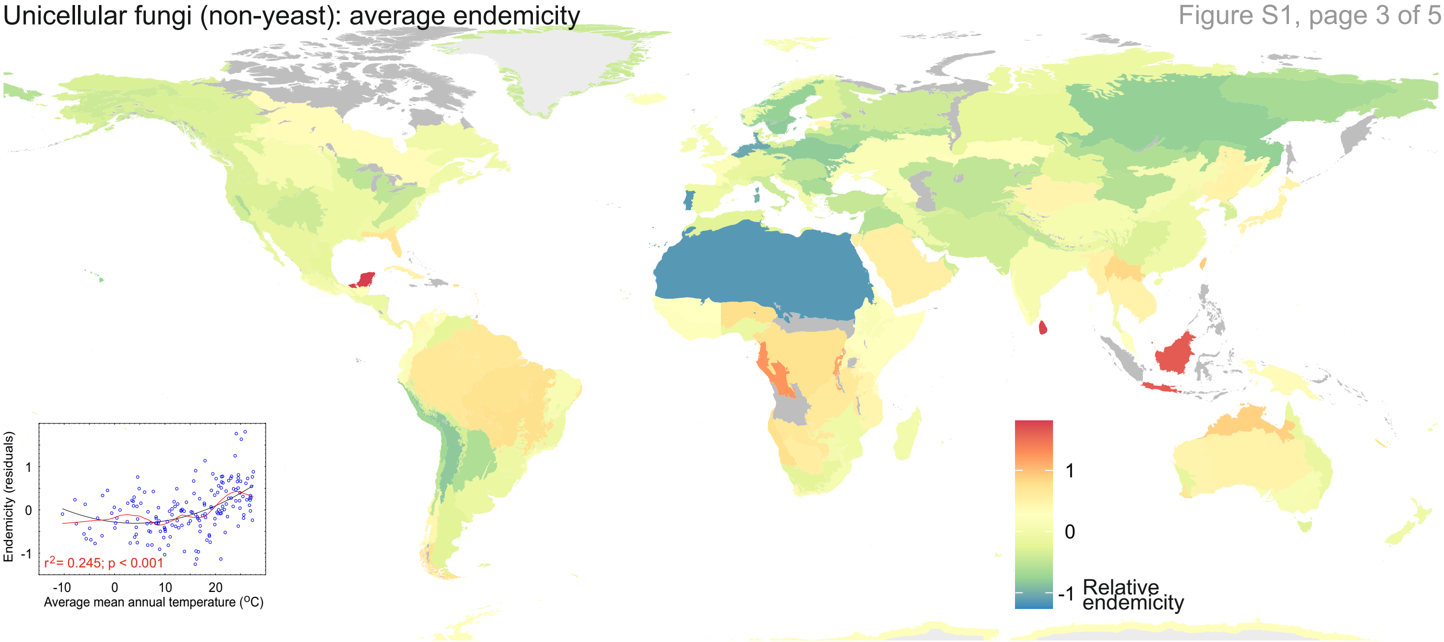

Yeasts: average endemicity

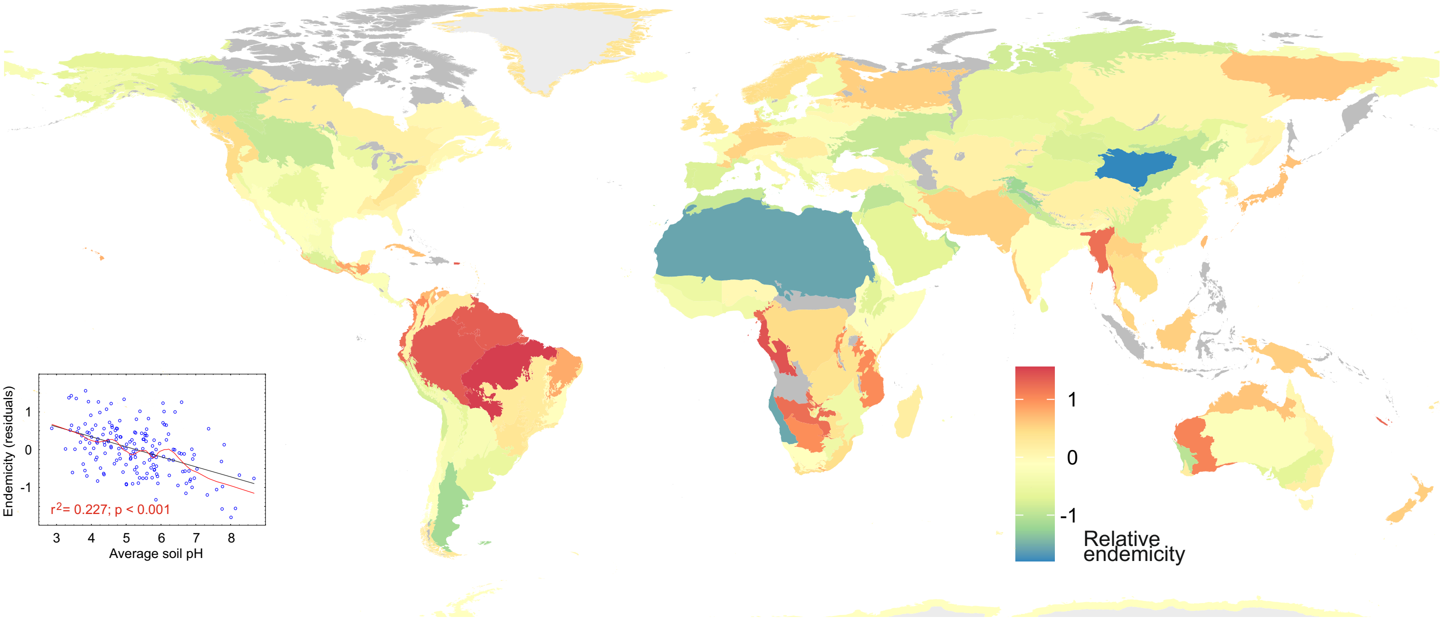

FIGURE S1 Average endemicity of fungal functional groups and five endemicity metrics of all fungi at the ecoregion scale. The insets indicate relationships to the best predictor (black line, best linear or quadratic fit; red line, lowess curve).

All fungi: mean geographic range of species

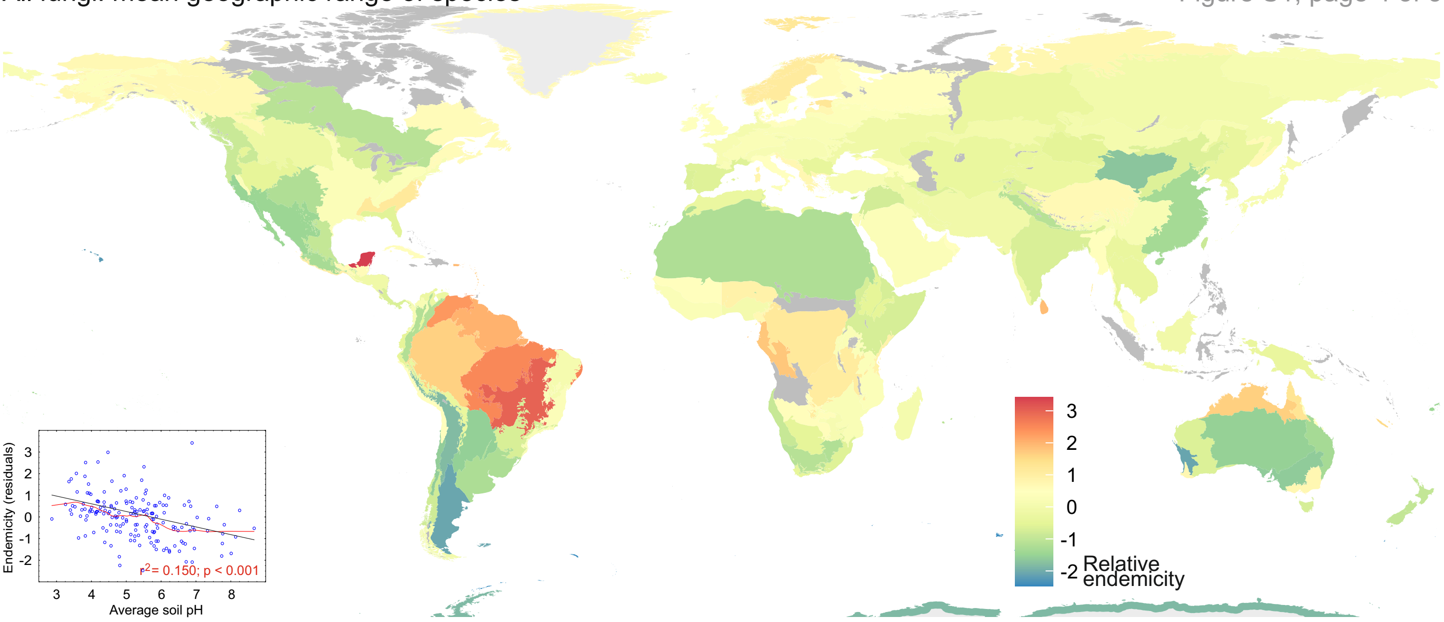

All fungi: number of endemic species

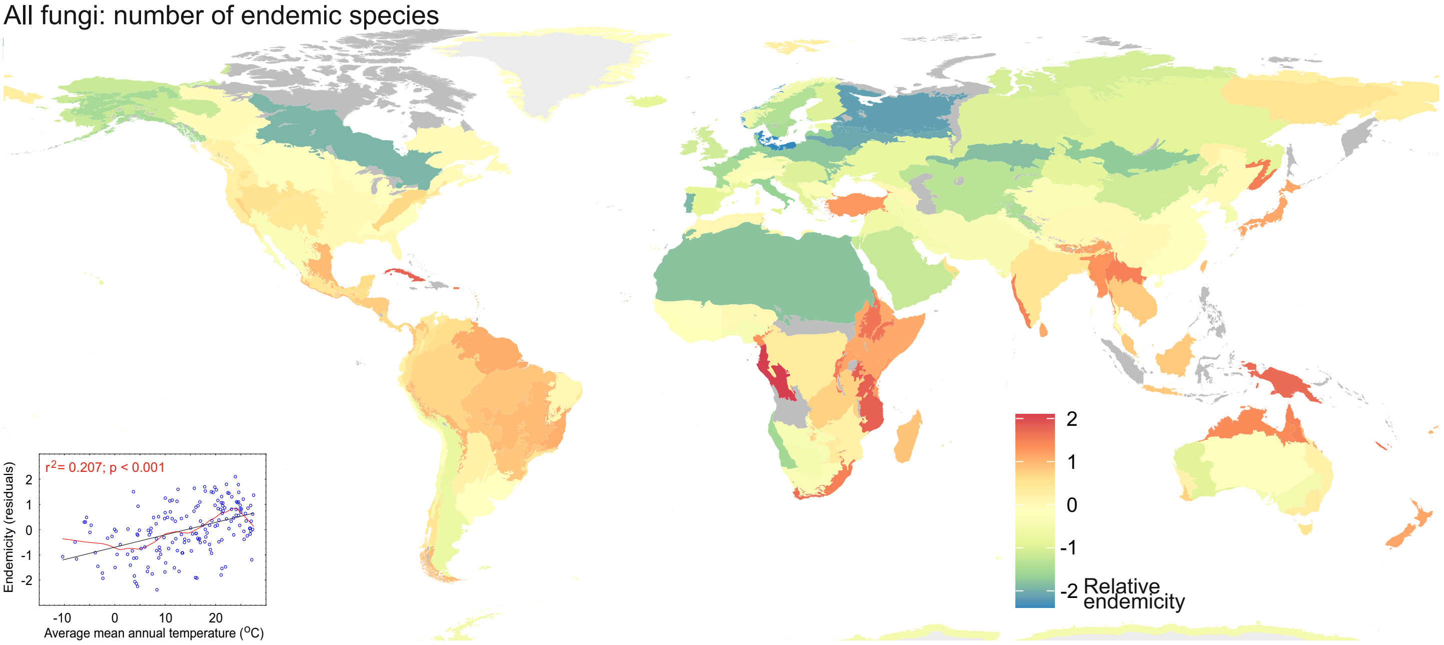

All fungi: proportion of endemic species

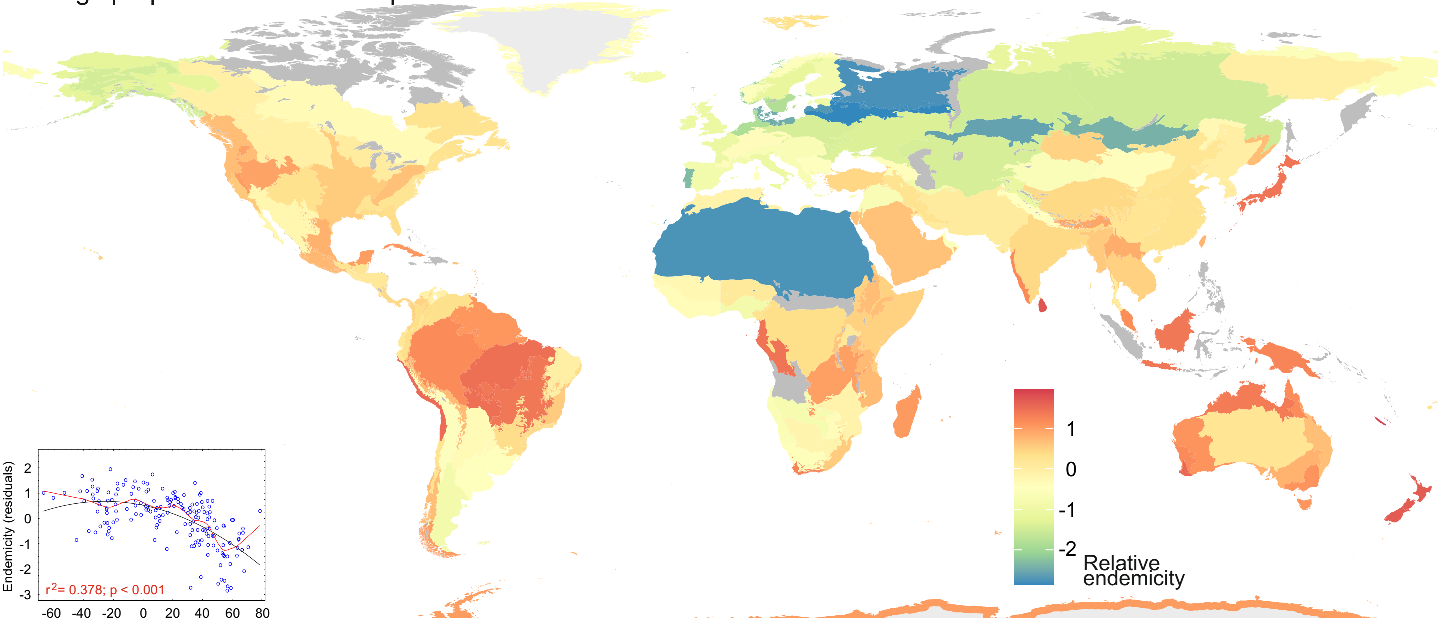

FIGURE S1 Average endemicity of fungal functional groups and five endemicity metrics of all fungi at the ecoregion scale. The insets indicate relationships to the best predictor (black line, best linear or quadratic fit; red line, lowess curve).

All fungi: average Jaccard distance

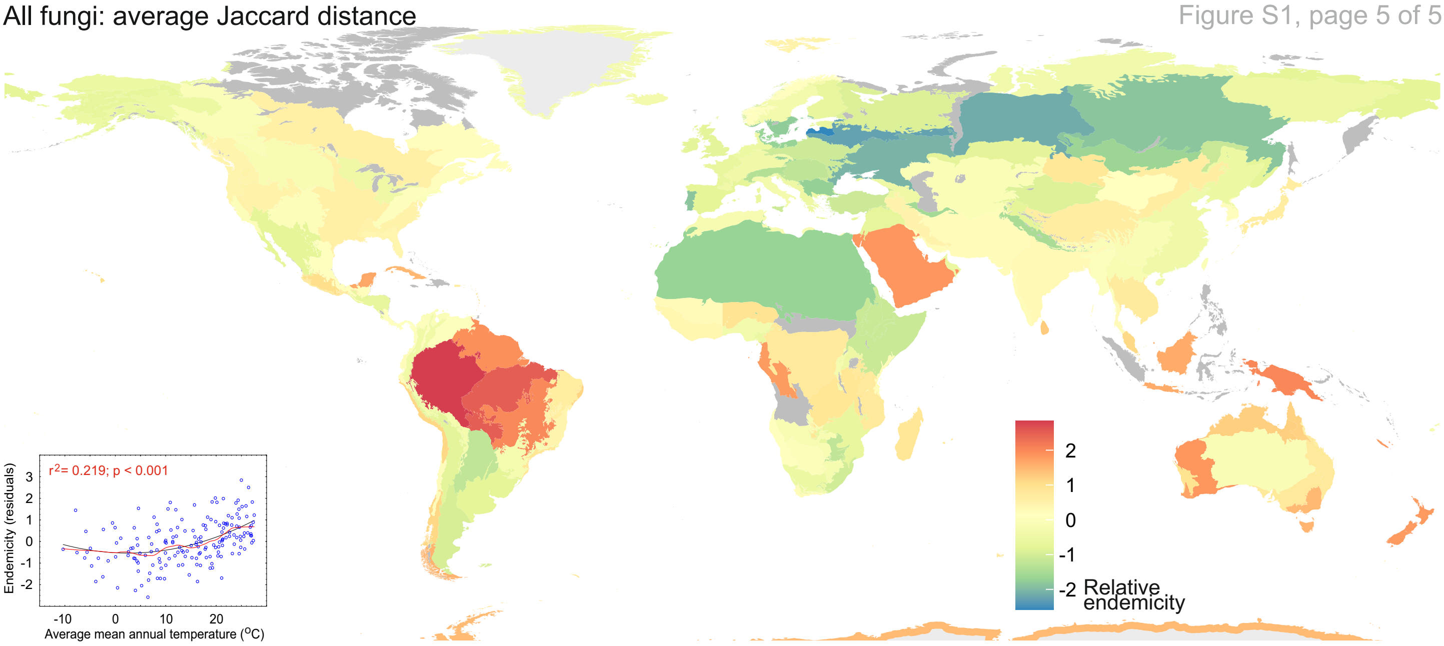

All fungi: average β-SIM (Simpson) distance

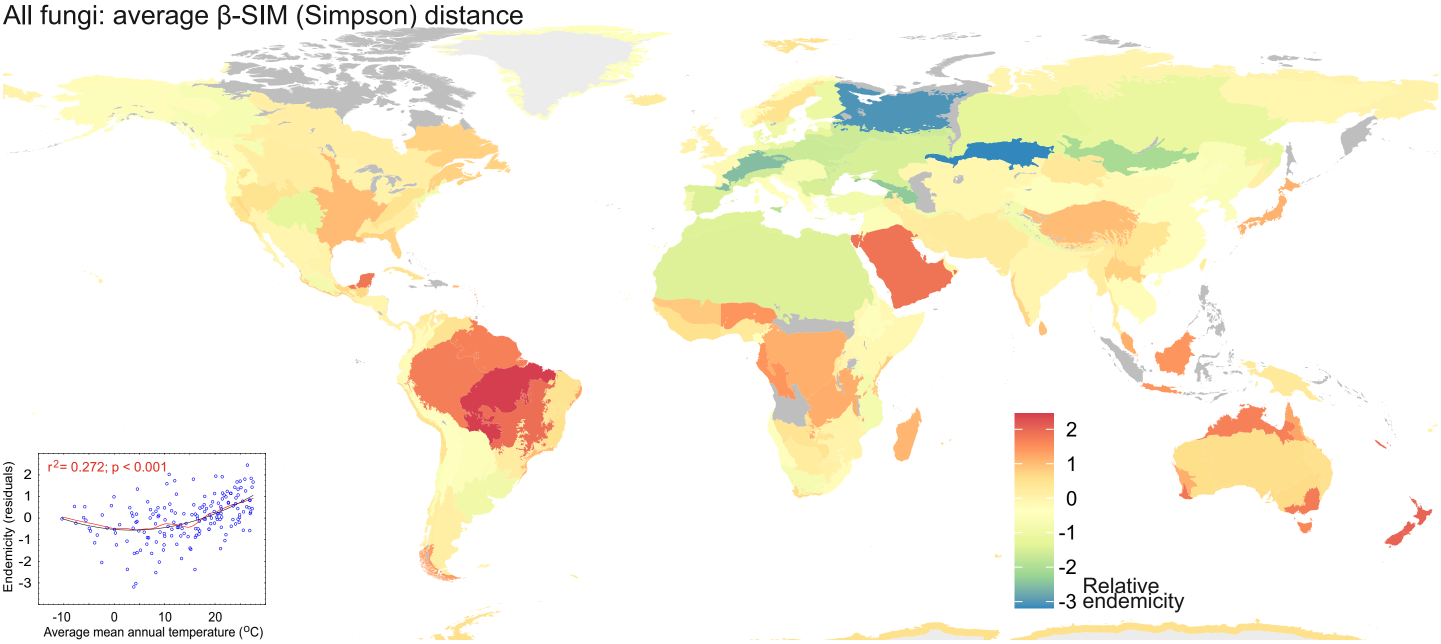

FIGURE S1 Average endemicity of fungal functional groups and five endemicity metrics of all fungi at the ecoregion scale. The insets indicate relationships to the best predictor (black line, best linear or quadratic fit; red line, lowess curve).

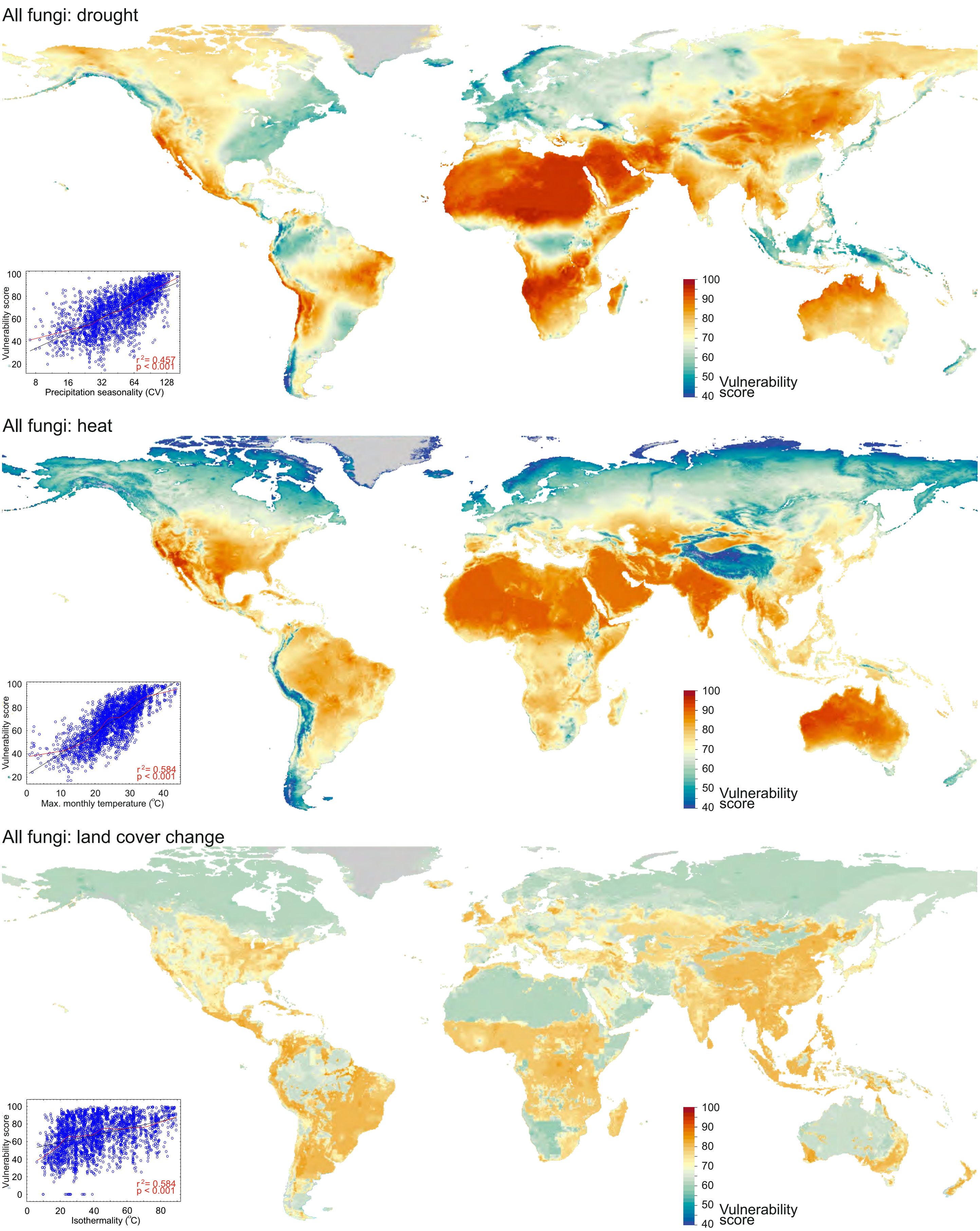

Figure S2 Vulnerability of all fungi to drought, heat, and land cover change based on cokriging. Insets indicate relationships to the best predictor. Error estimates are given in Figure S4.

Non-ectomycorrhizal Agaricomycetes: average vulnerability

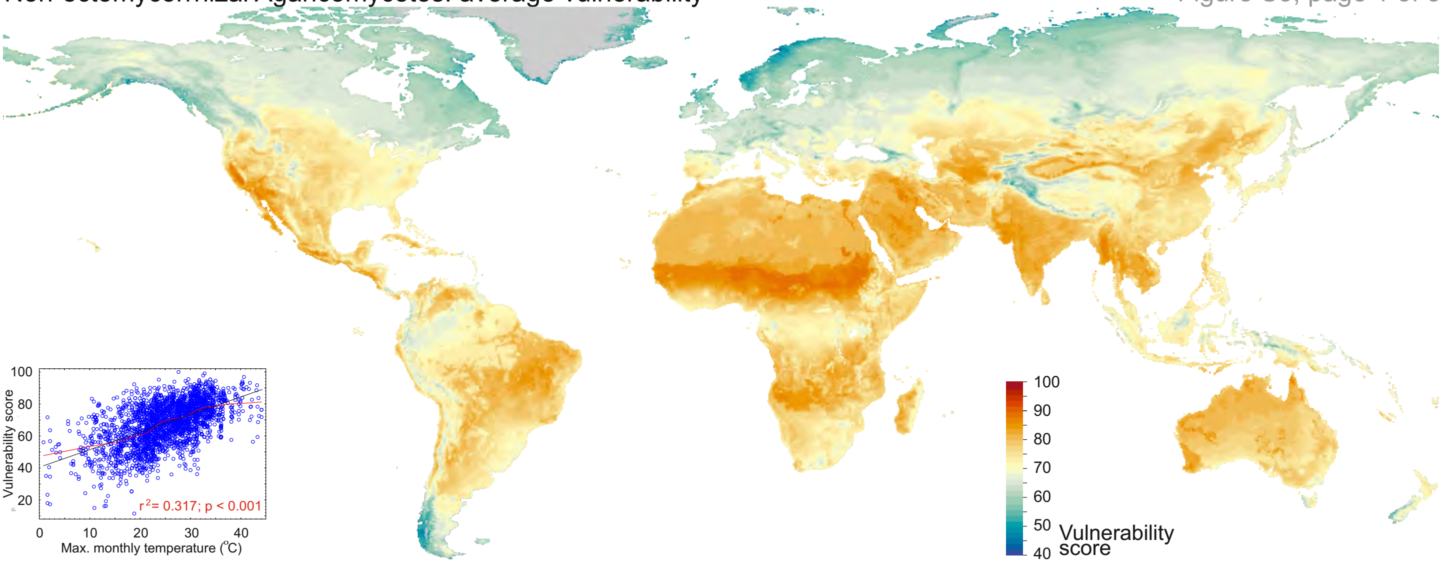

Arbuscular mycorrhizal fungi (Glomeromycota only): average vulnerability

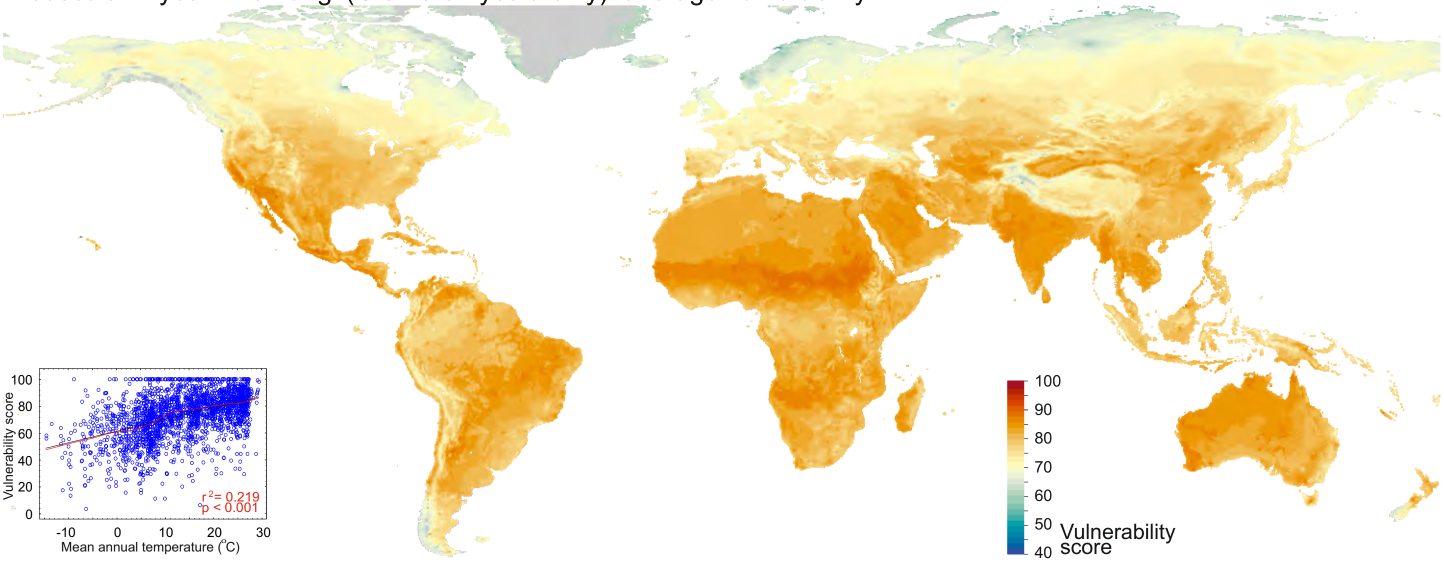

Ectomycorrhizal fungi: average vulnerability

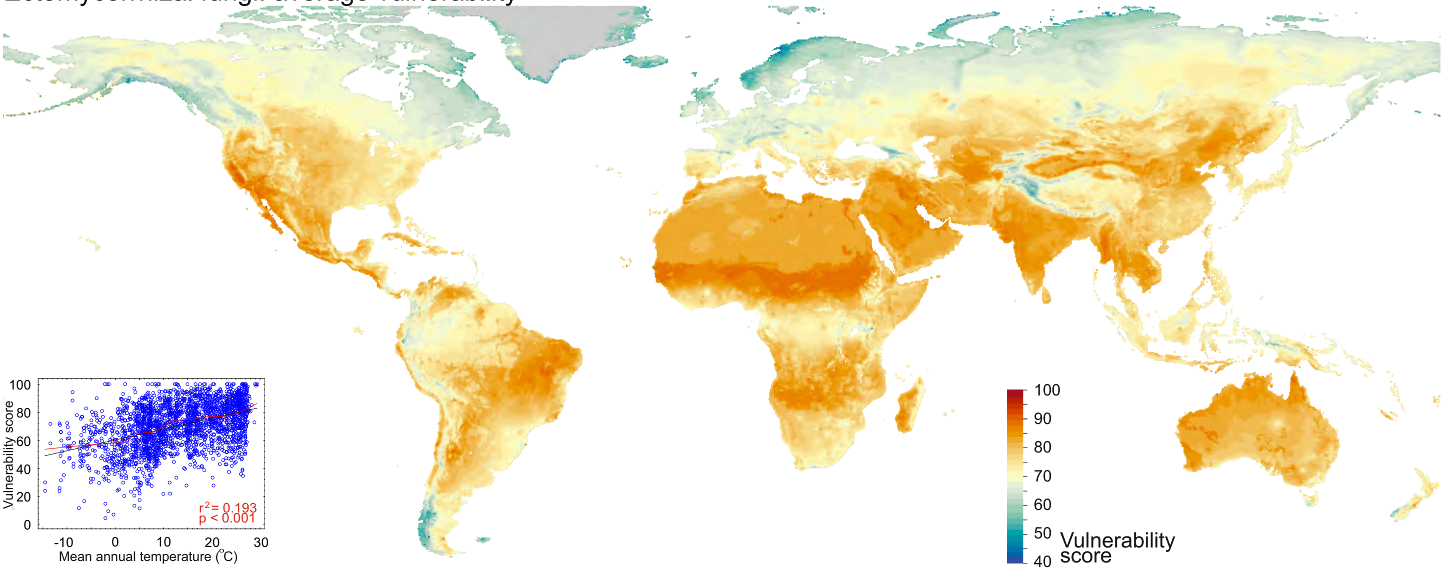

FIGURE S3 Average vulnerability of fungal functional groups to drought, heat, and land cover change based on cokriging. The insets indicate relationships to the best predictor. Error estimates are given in Figure S4.

Molds: average vulnerability

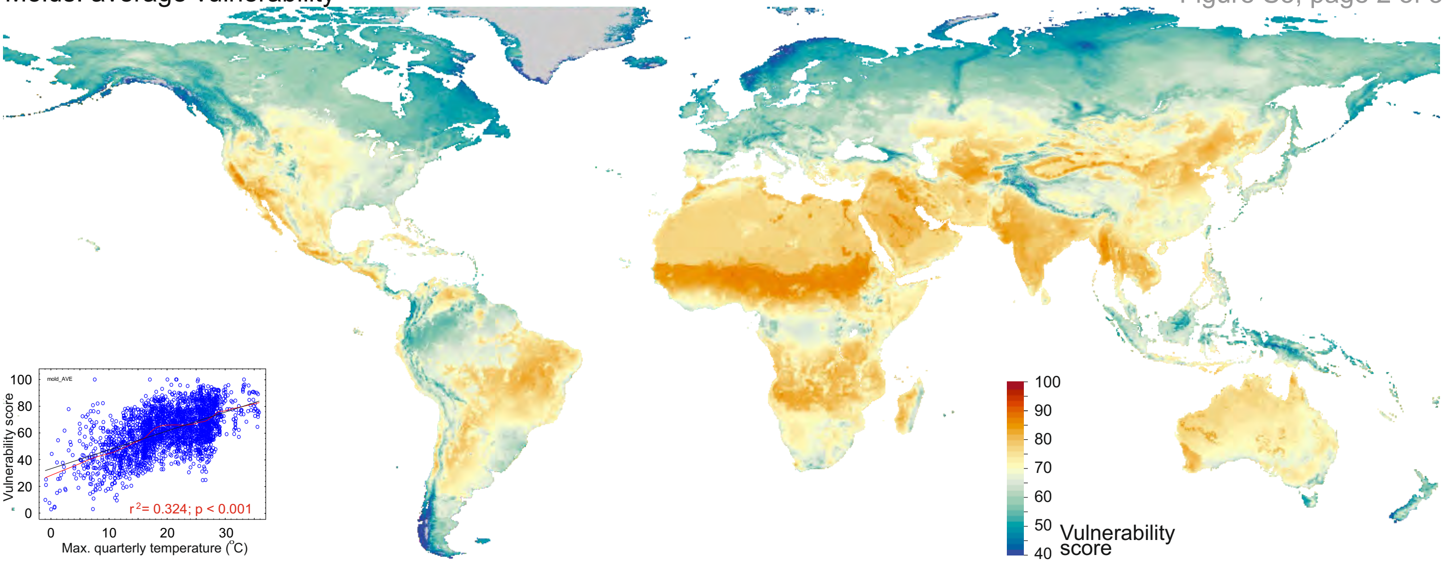

Opportunistic human parasites: average vulnerability

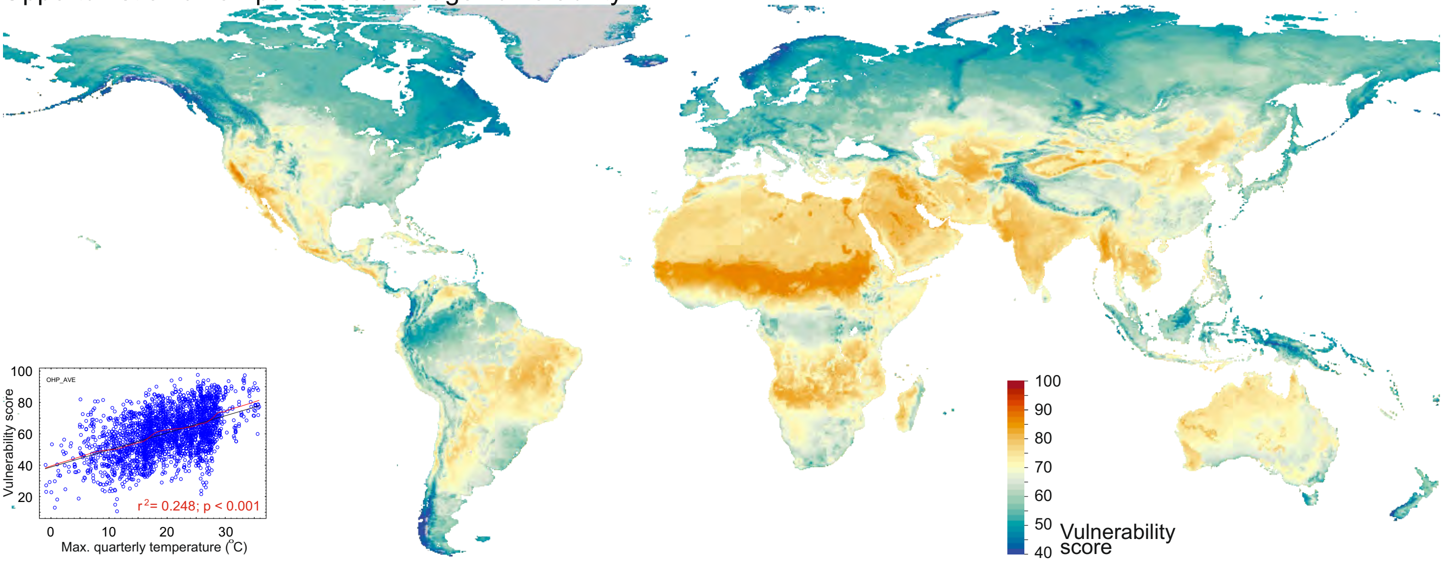

Pathogens: average vulnerability

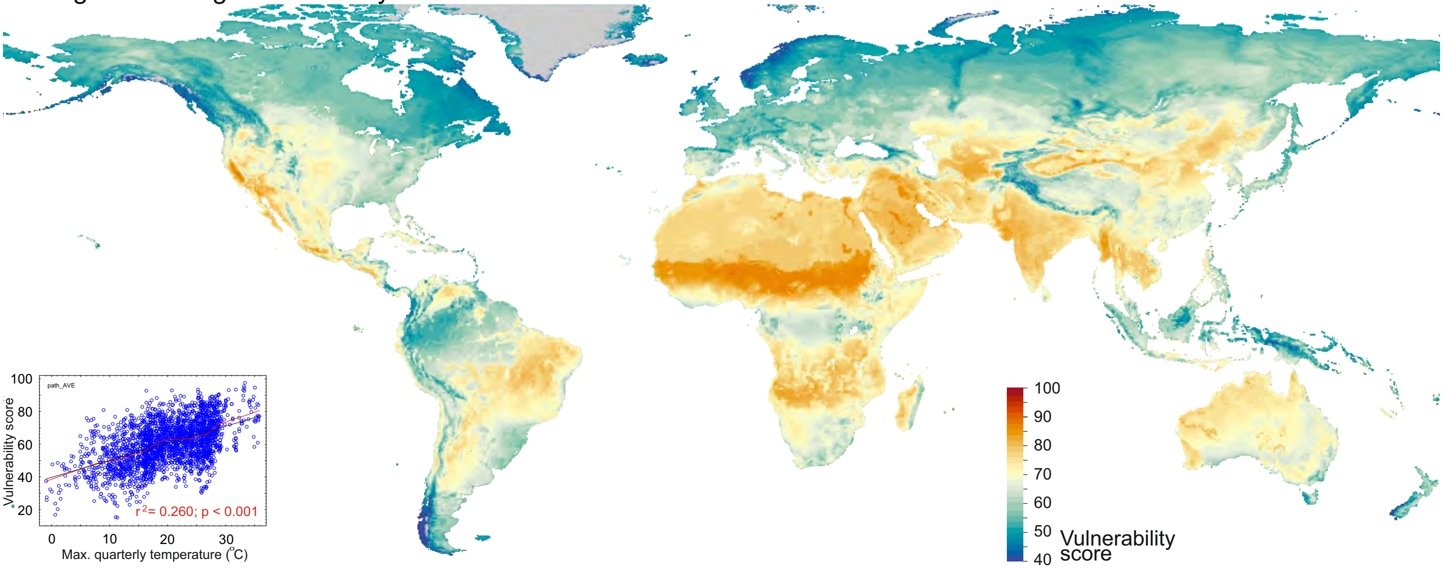

FIGURE S3 Average vulnerability of fungal functional groups to drought, heat, and land cover change based on cokriging. The insets indicate relationships to the best predictor. Error estimates are given in Figure S4.

Unicellular (non-yeast) fungi: average vulnerability

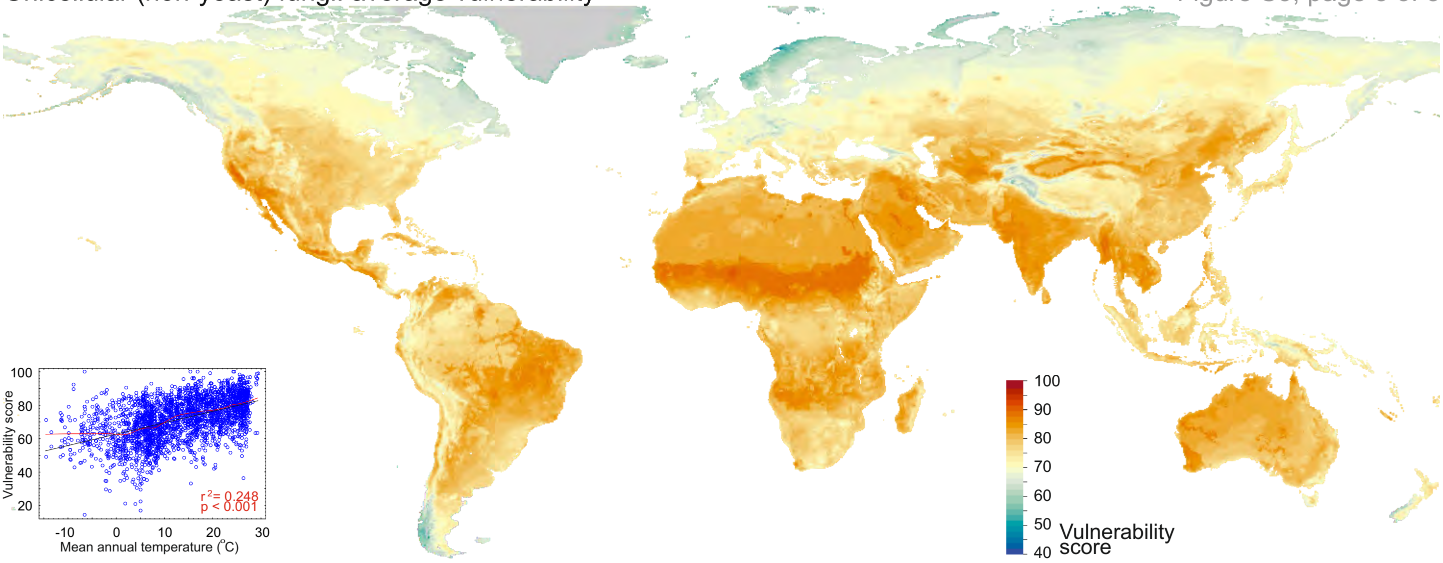

Yeasts: average vulnerability

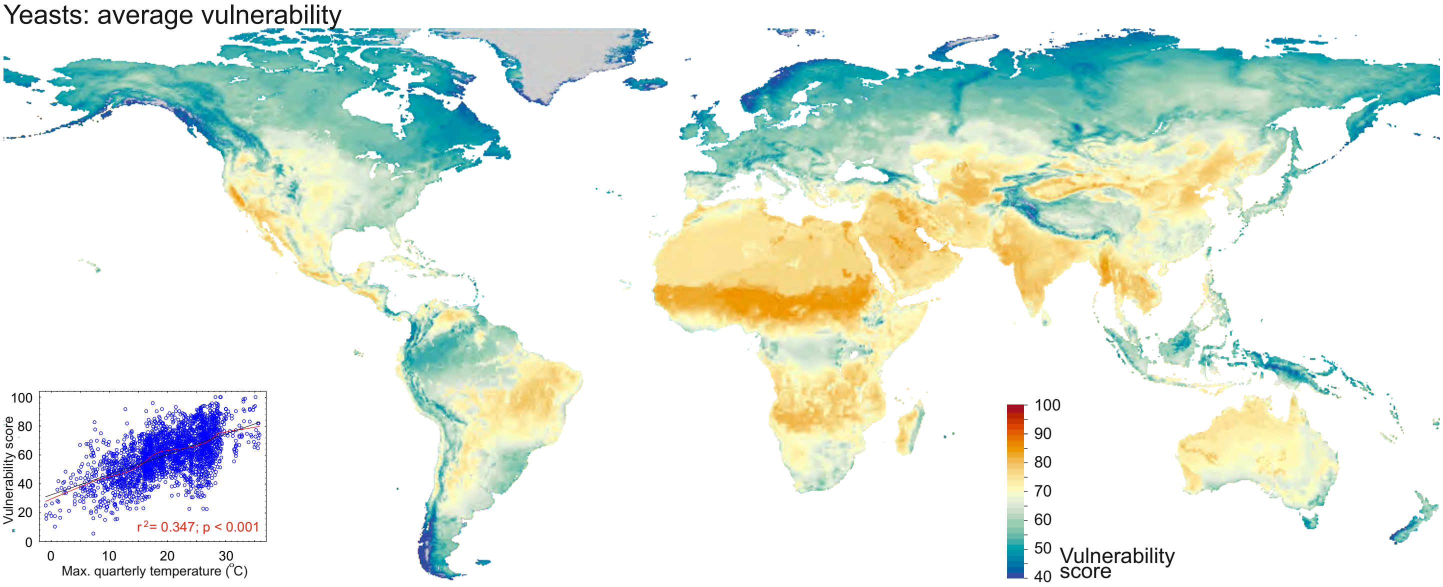

FIGURE S3 Average vulnerability of fungal functional groups to drought, heat, and land cover change based on cokriging. The insets indicate relationships to the best predictor. Error estimates are given in Figure S4.

All fungi: error estimates of average vulnerability

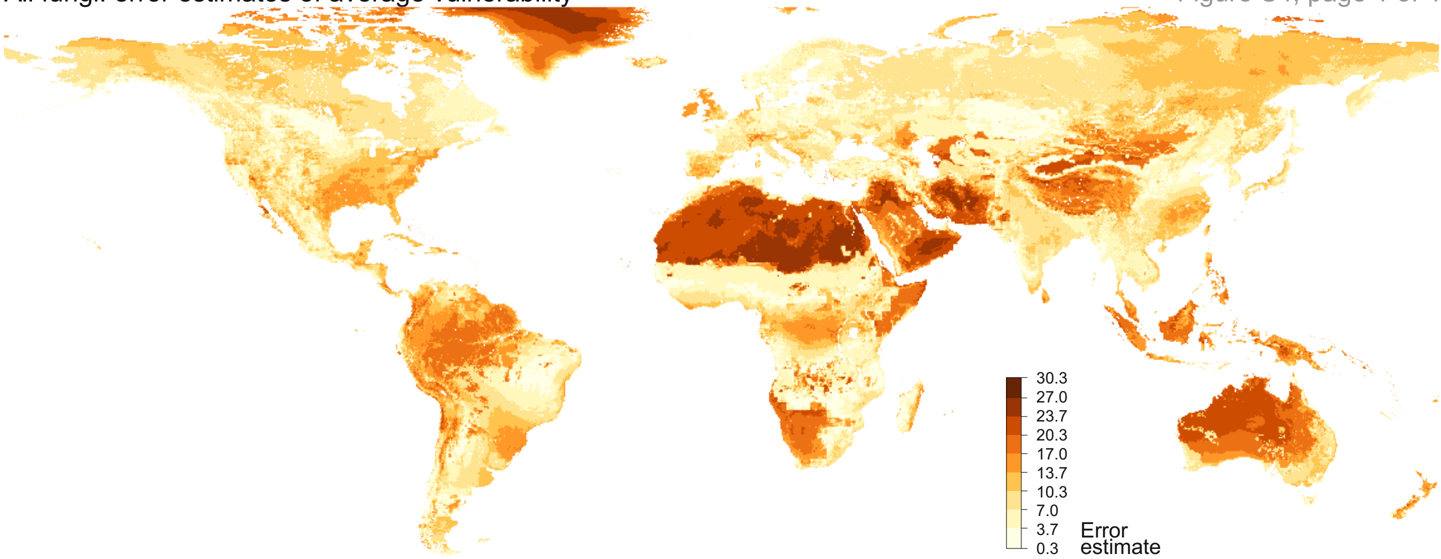

Non-ectomycorrhizal Agaricomycetes: error estimates of average vulnerability

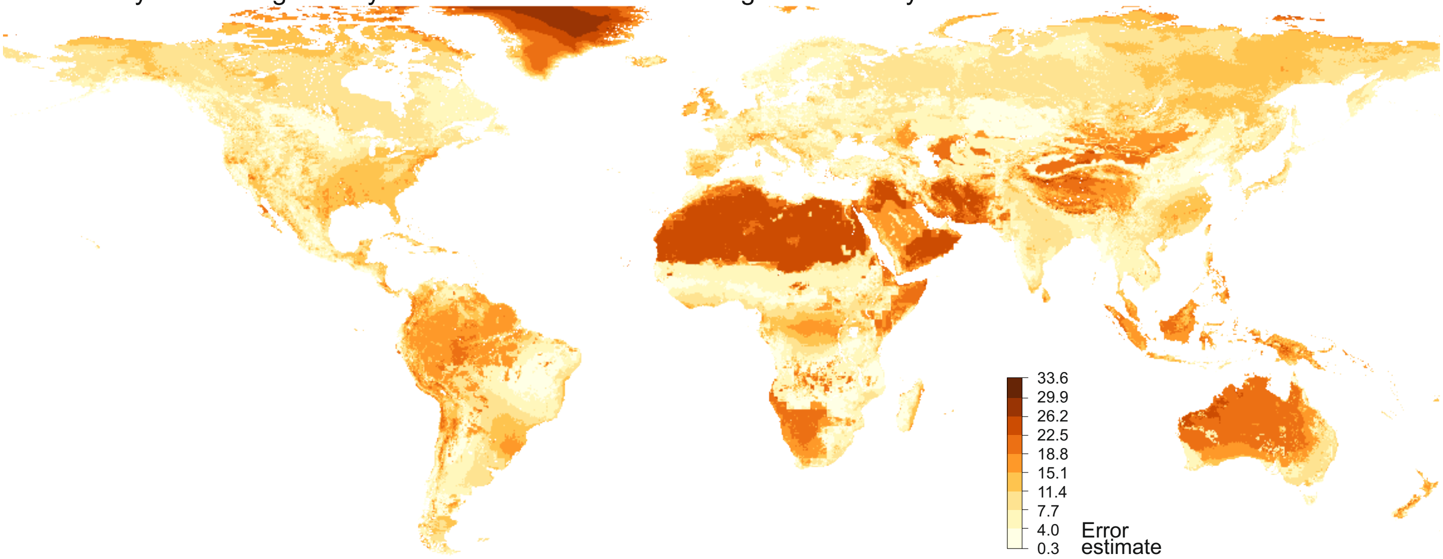

Arbuscular mycorrhizal fungi (Glomeromycota only): error estimates of average vulnerability

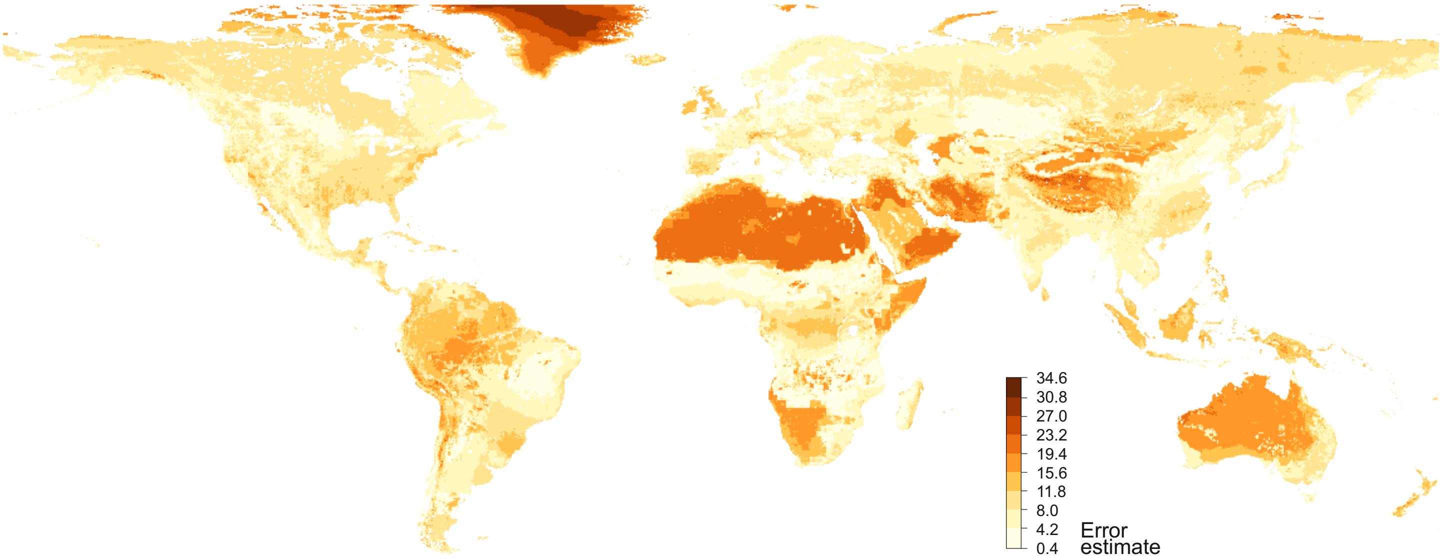

FIGURE S4 Error estimates of vulnerability of all fungi and fungal functional groups.

Ectomycorrhizal fungi: error estimates of average vulnerability

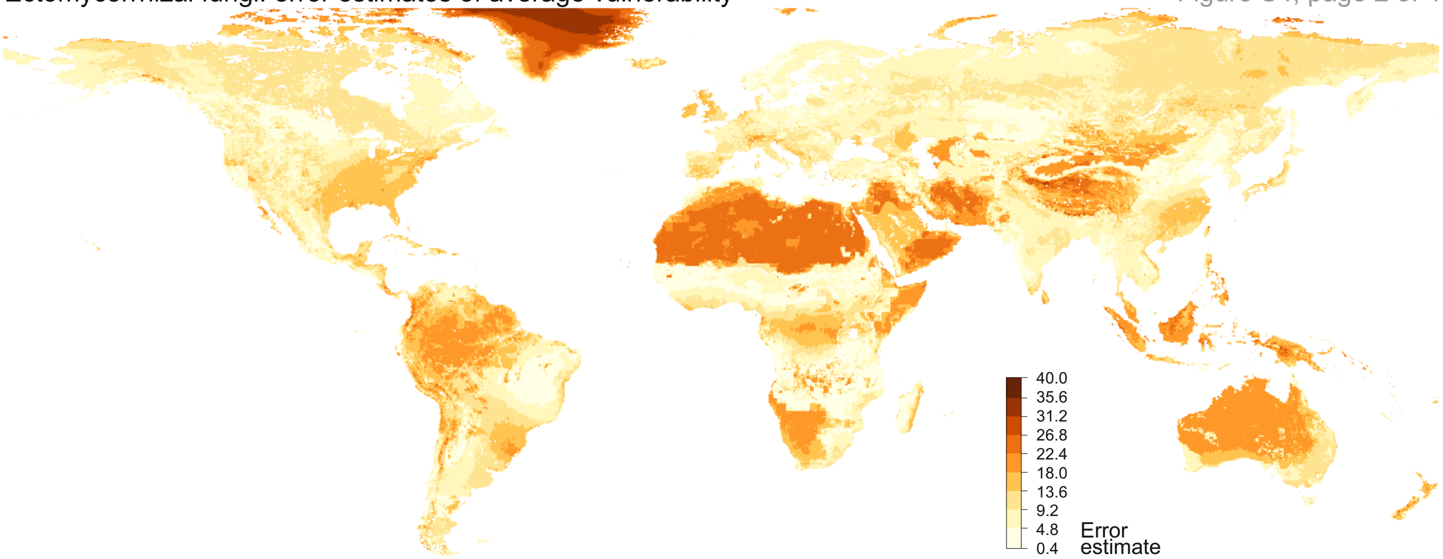

Molds: error estimates of average vulnerability

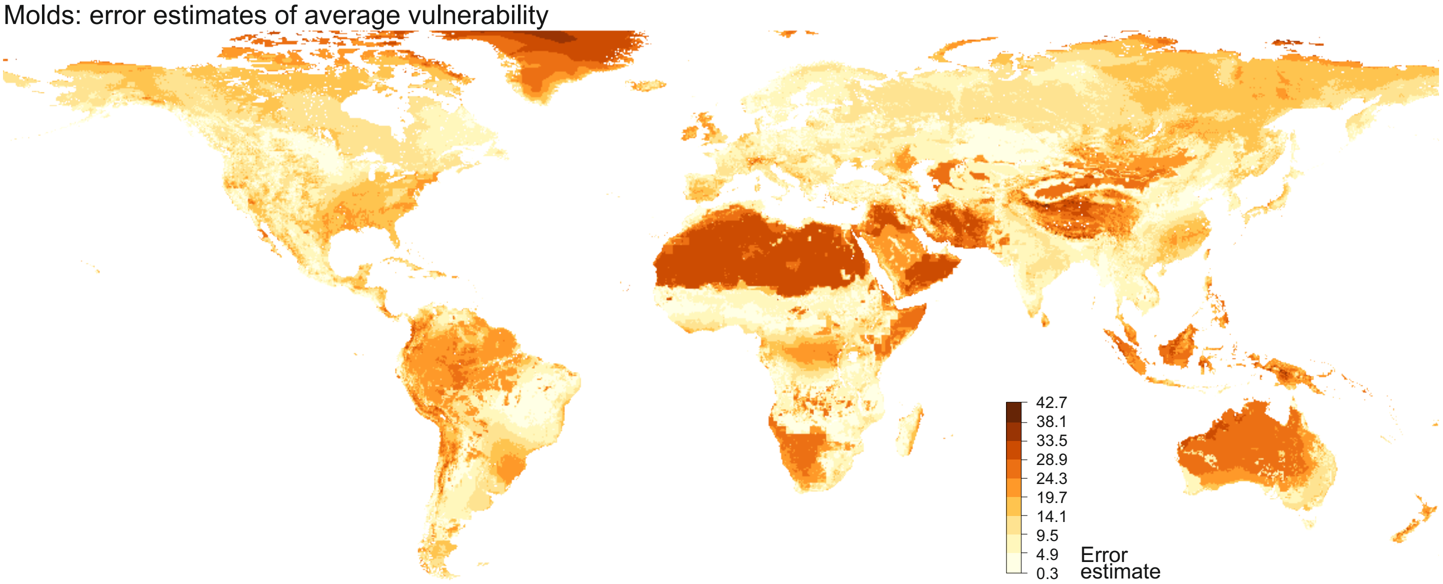

Opportunistic human parasites: error estimates of average vulnerability

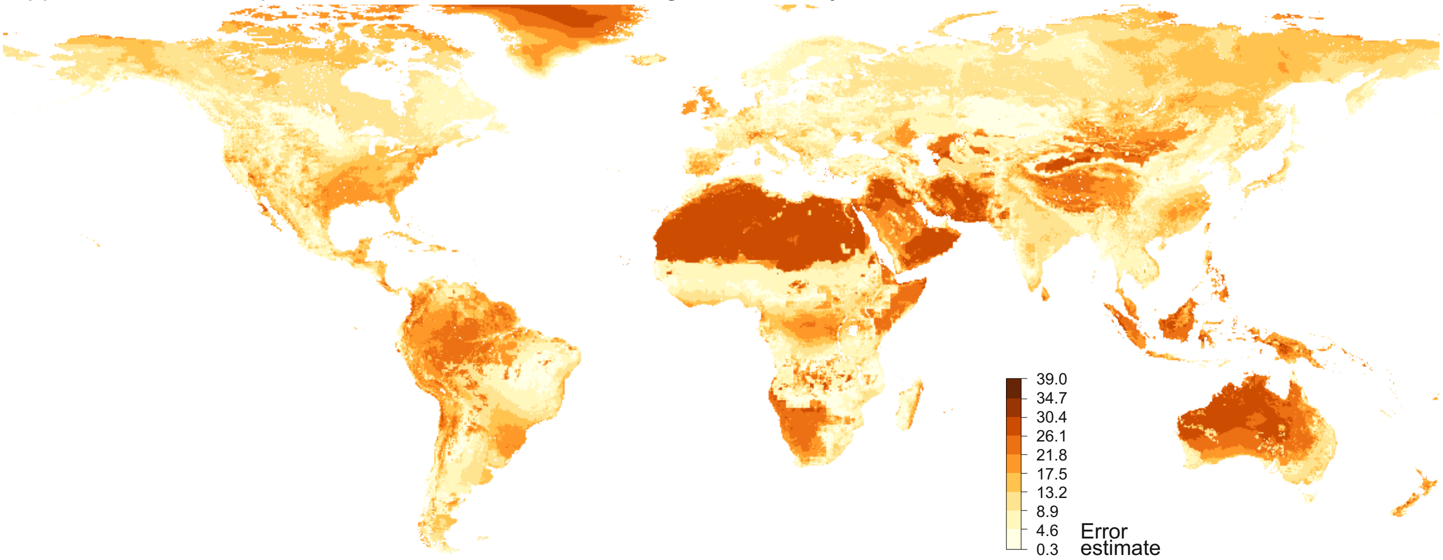

FIGURE S4 Error estimates of vulnerability of all fungi and fungal functional groups.

Pathogens: error estimates of average vulnerability

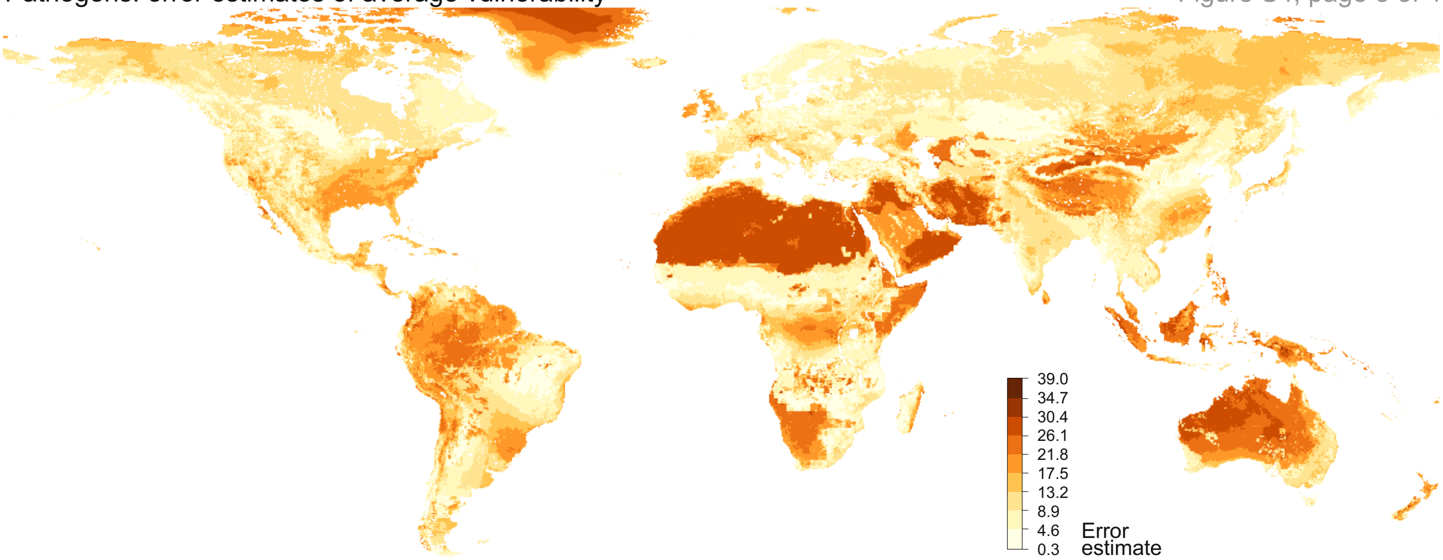

Unicellular (non-yeast) fungi: error estimates of average vulnerability

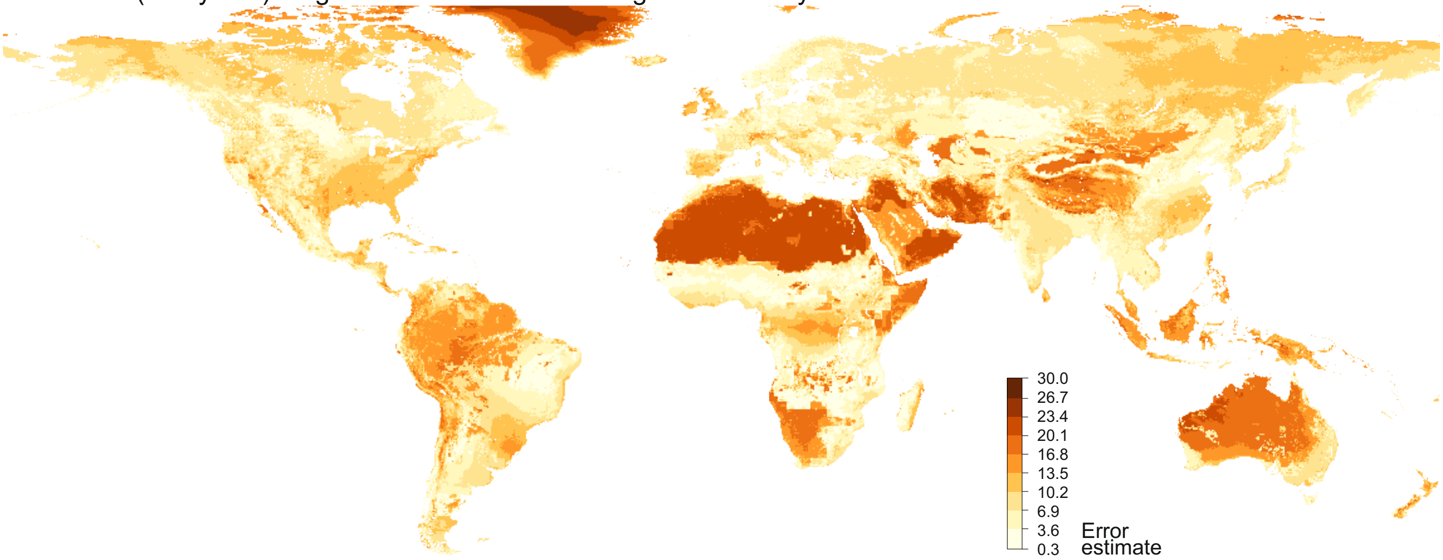

Yeasts: error estimates of average vulnerability

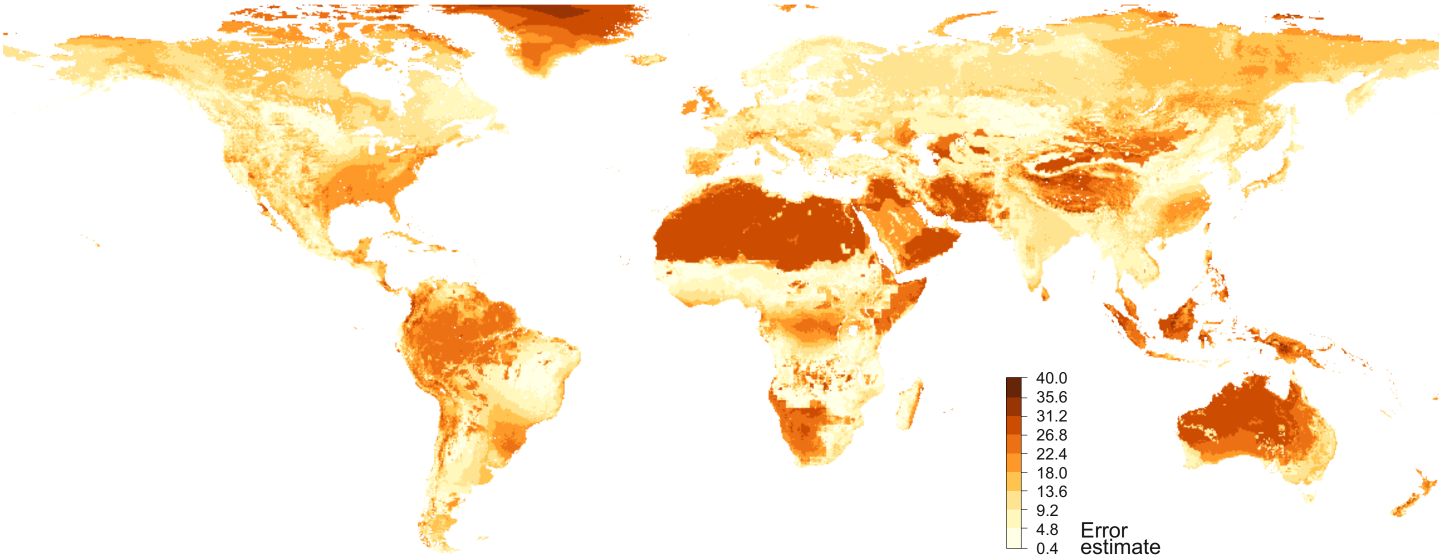

FIGURE S4 Error estimates of vulnerability of all fungi and fungal functional groups.

All fungi: error estimates of vulnerability to drought

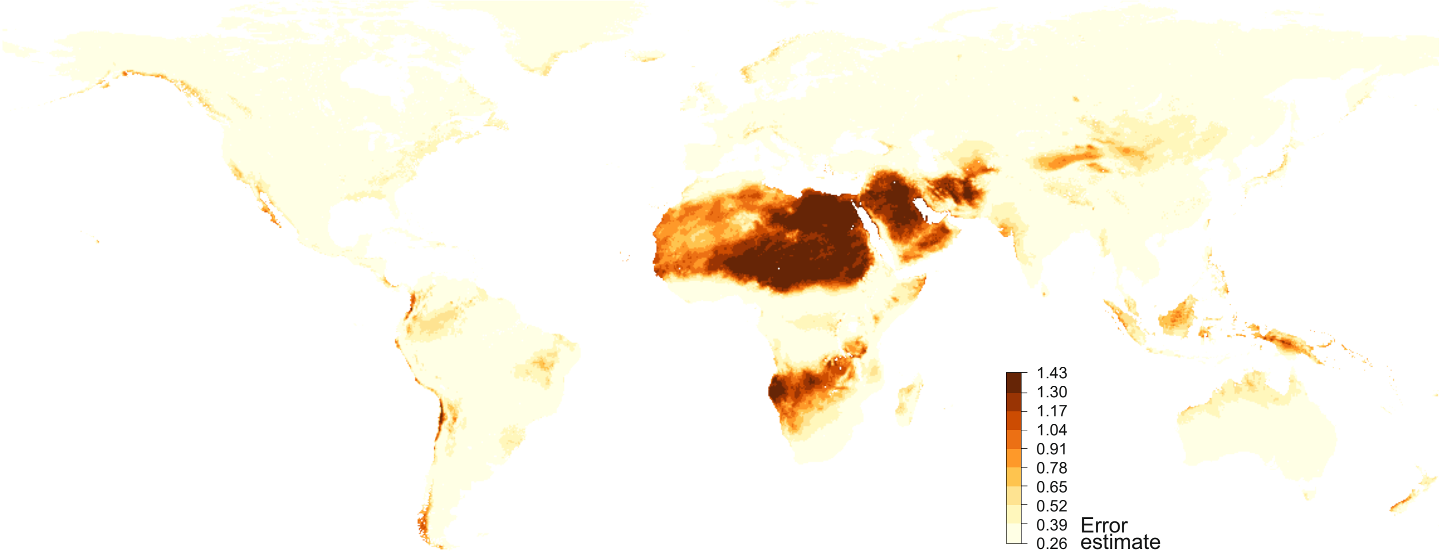

All fungi: error estimates of vulnerability to heat

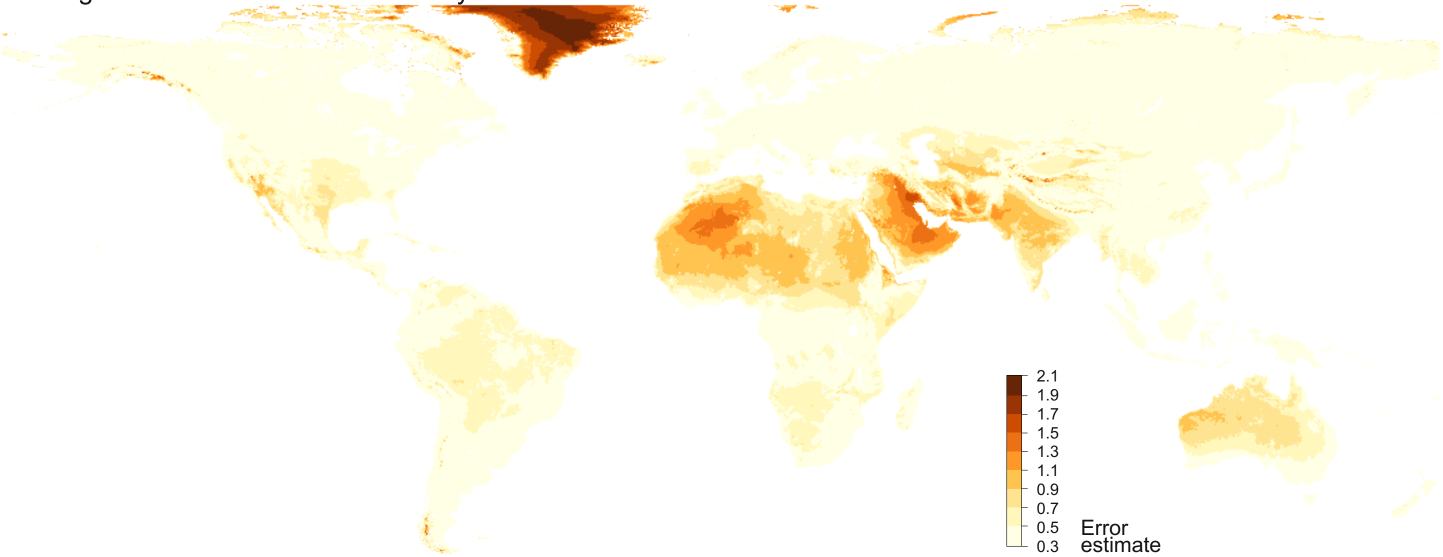

All fungi: error estimates of vulnerability to land cover change

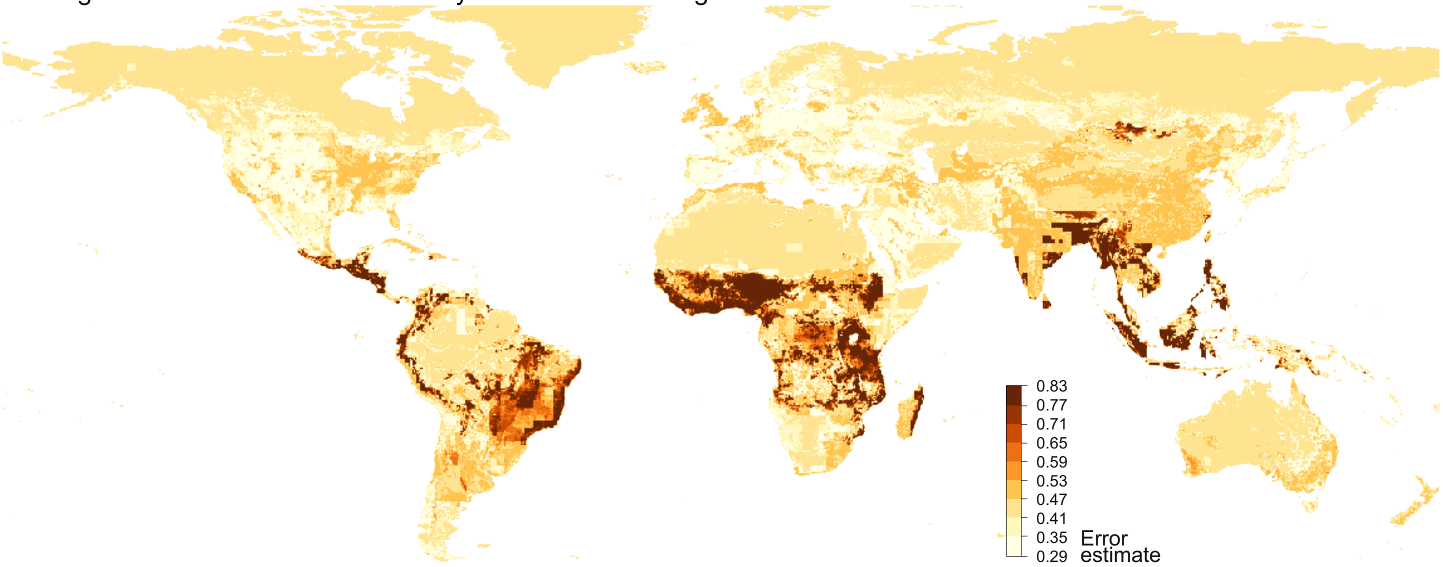

FIGURE S4 Error estimates of vulnerability of all fungi and fungal functional groups.
