# Supplementary material for: Global patterns in endemicity and vulnerability of soil fungi
Source: Glob Chang Biol. 2022 Sep 2;28(22):6696–710. doi: 10.1111/gcb.16398 (PMC9826061; doi:10.1111/gcb.16398)
Supplement: Supplementary file 5 — Table S3 [file GCB-28-6696-s004.docx]

**TABLE S3** The best models of conservation priority cokriging maps for fungi and functional groups. All P-values are <0.001.

|  | Df | Sum of squares | Mean squares | F-value | R^2^_adj_ | Trend |
| --- | --- | --- | --- | --- | --- | --- |
| **All fungi** |  |  |  |  |  |  |
| air MAT^1^ | 2 | 2133 | 1066 | 1426 | 0.266 | positive |
| MPWM | 1 | 992 | 992 | 663 | 0.134 | positive |
| climate isothermality | 1 | 169 | 169 | 113 | 0.023 | positive |
| MAP | 2 | 167 | 84 | 112 | 0.022 | positive |
| Land cover type^2^ | 7 | 128 | 18 | 12 | 0.015 |  |
| Land cover type x MAP | 7 | 121 | 17 | 12 | 0.014 |  |
| error | 2477 | 3705 |  |  |  |  |
| ^1^abbreviations: MAP, mean annual precipitation; MAT, mean annual temperature; MPWM, mean precipitation of wettest month.  ^2^Copernicus land cover types: broadleaf forest, mixed forest, coniferous forest, shrubland, tundra, grassland, desert, wetland (note that cropland and urban and village biomes were excluded). | | | | | | |

**TABLE S4** The best predictors of endemicity indices in ecoregions for all fungi and functional groups.

|  | DF | Sum of squares | Mean squares | F-value | R^2^_adj_ | P-value | Trend |
| --- | --- | --- | --- | --- | --- | --- | --- |
| **All fungi: average endemicity** | | | | | | | |
| MAT_mean_^1^ | 2 | 27.2 | 13.6 | 97.4 | 0.277 | <0.001 | U-shaped |
| soil pH_mean_ | 1 | 10.6 | 10.6 | 38.2 | 0.108 | <0.001 | negative |
| subcontinent^2^: Europe | 1 | 6.5 | 6.5 | 23.5 | 0.065 | <0.001 | negative |
| human footprint index | 1 | 2.1 | 2.1 | 7.5 | 0.018 | 0.007 | negative |
| error | 168 | 48.3 |  |  |  |  |  |
| **Agaricomycetes (non-mycorrhizal): average endemicity** | | | | | | | |
| MAT_mean_ | 1 | 33.2 | 33.2 | 98.1 | 0.308 | <0.001 | negative |
| soil pH_mean_ | 1 | 10.9 | 10.9 | 33.2 | 0.098 | <0.001 | positive |
| latitude | 1 | 4.4 | 4.4 | 13.1 | 0.038 | <0.001 | negative |
| error | 170 | 57.6 |  |  |  |  |  |
| **Arbuscular mycorrhizal (Glomeromycota): average endemicity** | | | | | | | |
| MAT_mean_ | 1 | 42.0 | 42.0 | 176.7 | 0.478 | <0.001 | positive |
| MAP_mean_ | 1 | 4.4 | 4.4 | 18.5 | 0.046 | <0.001 | positive |
| error | 171 | 40.7 |  |  |  |  |  |
| **Ectomycorrhizal fungi: average endemicity** | | | | | | | |
| MAP_mean_ | 1 | 3.8 | 3.8 | 31.4 | 0.142 | <0.001 | positive |
| Australia | 1 | 1.3 | 1.3 | 11.1 | 0.047 | <0.001 | positive |
| error | 171 | 20.6 |  |  |  |  |  |
| **Molds: average endemicity** | | | | | | | |
| soil pH_mean_ | 2 | 20.3 | 10.2 | 55.3 | 0.199 | <0.001 | U-shaped |
| MAT_mean_ | 1 | 12.3 | 12.3 | 33.9 | 0.121 | <0.001 | positive |
| LGM climate change | 1 | 4.9 | 4.9 | 15.0 | 0.047 | <0.001 | positive |
| LGM MDR_SD_ | 1 | 3.0 | 3.0 | 8.2 | 0.026 | 0.005 | positive |
| error | 169 | 62.1 |  |  |  |  |  |
| **Opportunistic human parasites: average endemicity** | | | | | | | |
| soil pH_mean_ | 2 | 20.6 | 10.3 | 48.0 | 0.185 | <0.001 | neg. expo. |
| human footprint | 1 | 6.5 | 6.5 | 15.1 | 0.056 | <0.001 | positive |
| LGM isothermality_mean_ | 1 | 3.7 | 3.7 | 8.6 | 0.030 | 0.004 | positive |
| LGM MDR_mean_ | 1 | 3.3 | 3.3 | 7.7 | 0.027 | 0.006 | positive |
| error | 168 | 72.2 |  |  |  |  |  |
| **Pathogens: average endemicity** | | | | | | | |
| soil pH_mean_ | 2 | 8.1 | 4.1 | 24.8 | 0.105 | <0.001 | U-shaped |
| subcontinent: Europe | 1 | 6.4 | 6.4 | 19.6 | 0.086 | <0.001 | negative |
| human footprint | 1 | 1.4 | 1.4 | 4.2 | 0.015 | 0.042 | positive |
| error | 171 | 57.3 |  |  |  |  |  |
| **Unicellular (non-yeast) fungi: average endemicity** | | | | | | | |
| MAT_mean_ | 2 | 12.4 | 6.2 | 61.6 | 0.236 | <0.001 | neg. expo. |
| soil pH_mean_ | 1 | 2.2 | 2.2 | 11.0 | 0.039 | 0.001 | negative |
| subcontinent: Europe | 1 | 2.0 | 2.0 | 10.0 | 0.035 | 0.002 | negative |
| error | 169 | 33.9 |  |  |  |  |  |
| **Yeasts: average endemicity** | | | | | | | |
| soil pH_mean_ | 1 | 16.6 | 16.6 | 62.8 | 0.222 | <0.001 | positive |
| MAT_mean_ | 1 | 11.4 | 11.4 | 43.2 | 0.151 | <0.001 | negative |
| error | 171 | 45.1 |  |  |  |  |  |
| **All fungi: mean maximum distance index** | | | | | | | |
| soil pH_mean_ | 1 | 25.9 | 25.9 | 37.3 | 0.145 | <0.001 | negative |
| MAT_mean_ | 2 | 17.9 | 8.9 | 25.8 | 0.094 | <0.001 | U-shaped |
| latitudinal span | 1 | 8.4 | 8.4 | 12.1 | 0.044 | <0.001 | negative |
| PDM_current-LGM_ | 1 | 4.2 | 4.2 | 6.0 | 0.020 | 0.015 | positive |
| error | 169 | 123.9 |  |  |  |  |  |
| **All fungi: number of endemic species** | | | | | | | |
| MAT_mean_ | 1 | 35.8 | 35.8 | 75.2 | 0.202 | <0.001 | negative |
| subcontinent: Europe | 1 | 29.2 | 29.2 | 61.3 | 0.164 | <0.001 | negative |
| soil pH_mean_ | 2 | 21.3 | 10.7 | 44.9 | 0.114 | <0.001 | unimodal |
| MAP_mean_ | 1 | 6.8 | 6.8 | 14.2 | 0.034 | <0.001 | positive |
| error | 171 | 79.5 |  |  |  |  |  |
| **All fungi: proportion of endemic species** | | | | | | | |
| latitude | 2 | 65.5 | 32.8 | 136.1 | 0.369 | <0.001 | negative |
| subcontinent: Europe | 1 | 12.6 | 20.6 | 26.1 | 0.068 | <0.001 | negative |
| soil pH_mean_ | 1 | 9.8 | 9.8 | 20.5 | 0.051 | <0.001 | negative |
| island habitat | 1 | 4.3 | 4.3 | 8.9 | 0.020 | 0.003 | positive |
| error | 168 | 80.8 |  |  |  |  |  |
| **All fungi: Jaccard distance index** | | | | | | | |
| MAT_mean_ | 2 | 37.8 | 18.9 | 51.0 | 0.209 | <0.001 | negative |
| latitude_mean_ | 1 | 17.4 | 17.4 | 30.3 | 0.096 | <0.001 | negative |
| environmental uniqueness | 1 | 12.6 | 12.6 | 22.0 | 0.068 | <0.001 | positive |
| soil pH_mean_ | 1 | 8.5 | 8.5 | 14.8 | 0.045 | <0.001 | negative |
| error | 168 | 96.6 |  |  |  |  |  |
| **All fungi: β-sim distance index** | | | | | | | |
| MAT_mean_ | 2 | 42.2 | 21.1 | 75.6 | 0.235 | <0.001 | U-shaped |
| latitude_mean_ | 1 | 17.3 | 17.3 | 30.9 | 0.095 | <0.001 | negative |
| soil pH_mean_ | 2 | 10.1 | 5.1 | 18.1 | 0.050 | <0.001 | neg. expo. |
| human footprint | 1 | 5.4 | 5.4 | 9.6 | 0.027 | 0.002 | negative |
| LGM climate change | 1 | 5.2 | 5.2 | 9.2 | 0.025 | 0.003 | positive |
| error | 166 | 86.8 |  |  |  |  |  |

^1^abbreviations: LGM, last glacial maximum; MAP, mean annual precipitation; MAT, mean annual temperature; MDR, mean diurnal range; PDM, precipitation of the driest month.

^2^subcontinents: Africa, Antarctica, Australia, Central America, Central Asia, East Asia, Europe, North America, South America, Oceania, South Asia, Southern South America.

**TABLE S5** Best predictors of global change vulnerability of all fungi and functional groups. These models were used for vulnerability regression-cokriging analyses.

|  | Df | Sum of squares | Mean squares | F-value | R^2^_adj_ | P-value | Trend |
| --- | --- | --- | --- | --- | --- | --- | --- |
| **All fungi: average vulnerability** | | | | | | | |
| MTWQ^1^ | 1 | 109467 | 109467 | 1216 | 0.274 | <0.001 | positive |
| climate isothermality | 1 | 16131 | 16131 | 179 | 0.040 | <0.001 | positive |
| MDR | 1 | 14916 | 14916 | 166 | 0.037 | <0.001 | positive |
| land cover type | 9 | 13713 | 1524 | 17 | 0.034 | <0.001 |  |
| land cover type x MDR | 9 | 12618 | 1402 | 16 | 0.031 | <0.001 |  |
| error | 2560 | 230517 |  |  |  |  |  |
| **All fungi: vulnerability to drought** | | | | | | | |
| precipitation seasonality | 1 | 350907 | 350907 | 2567 | 0.456 | <0.001 | positive |
| MPWM | 1 | 29003 | 29003 | 212 | 0.037 | <0.001 | positive |
| land cover type | 9 | 24154 | 2684 | 20 | 0.030 | <0.001 |  |
| land cover type x MPWM | 9 | 13068 | 1452 | 11 | 0.016 | <0.001 |  |
| error | 2561 | 350115 |  |  |  |  |  |
| **All fungi: vulnerability to heat** | |  |  |  |  |  |  |
| MTWM | 1 | 423545 | 423545 | 4399 | 0.583 | <0.001 | positive |
| MTWQ | 1 | 10119 | 10119 | 105 | 0.013 | <0.001 | positive |
| land cover type | 9 | 28522 | 3169 | 33 | 0.038 | <0.001 |  |
| land cover type x MTWM | 9 | 15892 | 1766 | 18 | 0.021 | <0.001 |  |
| error | 2561 | 246554 |  |  |  |  |  |
| **All fungi: vulnerability to land use change** | | | | | | | |
| climate isothermality | 1 | 128040 | 128040 | 451 | 0.135 | <0.001 | positive |
| human footprint index | 1 | 46462 | 46462 | 164 | 0.048 | <0.001 | positive |
| MTWM | 1 | 3958 | 3958 | 14 | 0.003 | <0.001 | positive |
| land cover type | 9 | 35033 | 3893 | 14 | 0.036 | <0.001 |  |
| land cover type x isothermality | 9 | 10763 | 1196 | 4 | 0.010 | <0.001 |  |
| land cover type x MTWM | 9 | 11986 | 1332 | 5 | 0.012 | <0.001 |  |
| error | 2494 | 708337 |  |  |  |  |  |
| **Agaricomycetes (non-mycorrhizal): average vulnerability** | | | | |  |  |  |
| MTWM | 1 | 150610 | 150610 | 1461 | 0.316 | <0.001 | positive |
| latitude | 2 | 32363 | 16181 | 314 | 0.066 | <0.001 | unimodal |
| air MAP | 1 | 957 | 957 | 9 | 0.001 | 0.002 | positive |
| land cover type | 9 | 17268 | 1919 | 19 | 0.035 | <0.001 |  |
| land cover type x air MAP | 9 | 10690 | 1188 | 12 | 0.022 | <0.001 |  |
| error | 2549 | 262695 |  |  |  |  |  |
| **Arbuscular mycorrhizal (Glomeromycota): average vulnerability** | | | | | |  |  |
| MAT | 1 | 141000 | 141000 | 718 | 0.218 | <0.001 | positive |
| MDR | 1 | 36490 | 36490 | 186 | 0.056 | <0.001 | positive |
| latitude | 2 | 15994 | 7997 | 81 | 0.023 | <0.001 | unimodal |
| land cover type | 9 | 10529 | 1170 | 6 | 0.015 | <0.001 |  |
| error | 2243 | 440722 |  |  |  |  |  |
| **Ectomycorrhizal fungi: average vulnerability** | | | |  |  |  |  |
| MAT | 1 | 124728 | 124728 | 695 | 0.191 | <0.001 | positive |
| MDR | 1 | 38656 | 38656 | 215 | 0.059 | <0.001 | positive |
| MTWQ | 1 | 10589 | 10589 | 59 | 0.015 | <0.001 | positive |
| land cover type | 9 | 26923 | 2991 | 17 | 0.041 | <0.001 |  |
| land cover type x MDR | 9 | 19693 | 2188 | 12 | 0.029 | <0.001 |  |
| error | 2383 | 427503 |  |  |  |  |  |
| **Molds: average vulnerability** | | |  |  |  |  |  |
| MTWQ | 1 | 214568 | 214568 | 1592 | 0.323 | <0.001 | positive |
| precipitation seasonality | 1 | 52259 | 52259 | 388 | 0.078 | <0.001 | positive |
| MTWM | 1 | 33034 | 33034 | 245 | 0.049 | <0.001 | positive |
| land cover type | 9 | 18316 | 2035 | 15 | 0.027 | <0.001 |  |
| error | 2546 | 343208 |  |  |  |  |  |
| **Opportunistic human pathogens: average vulnerability** | | | | |  |  |  |
| MTWQ | 1 | 129358 | 129358 | 1098 | 0.247 | <0.001 | positive |
| precipitation seasonality | 1 | 51490 | 51490 | 437 | 0.098 | <0.001 | positive |
| MTWM | 1 | 27118 | 27118 | 230 | 0.051 | <0.001 | positive |
| land cover type | 9 | 11809 | 1312 | 11 | 0.022 | <0.001 |  |
| error | 2564 | 302207 |  |  |  |  |  |
| **Pathogens: average vulnerability** | | |  |  |  |  |  |
| MTWQ | 1 | 118928 | 118928 | 1155 | 0.259 | <0.001 | positive |
| MDR | 1 | 47910 | 47910 | 465 | 0.104 | <0.001 | positive |
| land cover type | 9 | 11541 | 1282 | 12 | 0.024 | <0.001 |  |
| land cover type x MDR | 9 | 14857 | 1651 | 16 | 0.032 | <0.001 |  |
| error | 2559 | 263439 |  |  |  |  |  |
| **Unicellular (non-yeast) fungi: average vulnerability** | | | | |  |  |  |
| MAT | 1 | 103333 | 103333 | 1014 | 0.247 | <0.001 | positive |
| temperature annual range | 1 | 19071 | 19071 | 187 | 0.045 | <0.001 | negative |
| climate isothermality | 1 | 19913 | 19913 | 195 | 0.047 | <0.001 | positive |
| land cover type | 9 | 12752 | 1417 | 14 | 0.030 | <0.001 |  |
| error | 2560 | 260948 |  |  |  |  |  |
| **Yeasts: average vulnerability** | | |  |  |  |  |  |
| MTWQ | 1 | 211085 | 211085 | 1790 | 0.346 | <0.001 | positive |
| MDR | 1 | 43113 | 43113 | 366 | 0.070 | <0.001 | positive |
| precipitation seasonality | 1 | 30172 | 30172 | 256 | 0.049 | <0.001 | positive |
| land cover type | 9 | 23685 | 2632 | 22 | 0.038 | <0.001 |  |
| error | 2541 | 299600 |  |  |  |  |  |

^1^abbreviations: MAP, mean annual precipitation; MAT, mean annual temperature; MDR, mean diurnal range; MPWM, mean precipitation of wettest month; MTWM, mean temperature of warmest month; MTWQ, mean temperature of warmest quarter.

^2^Copernicus land cover types: broadleaf forest, mixed forest, coniferous forest, shrubland, tundra, grassland, desert, wetland, cropland, and urban and village.

**TABLE S6** Additional funding sources by authors.

| Researcher | Funding acknowledgements |
| --- | --- |
| Leho Tedersoo | Estonian Science Foundation (grants PRG632, MOBTP198), EEA Financial Mechanism Baltic Research Programme (EMP442), King Saud University DFSP-2020-2 and Novo Nordisk Fonden (NNF20OC0059948). |
| Vladimir Mikryukov | Estonian Science Foundation grant PRG632 |
| Mohammad Bahram | Estonian Science Foundation grant PRG632 |
| Niloufar Hagh-Doust | Estonian Science Foundation grant PRG632 |
| Sten Anslan | Estonian Science Foundation (grants PRG632, MOBTP198) |
| Manuel Delgado-Baquerizo | British Ecological Society (grant agreement No LRB17\1019 [MUSGONET]), Spanish Ministry of Science and Innovation (PID2020-115813RA-I00 [SOIL4GROWTH] funded by MCIN/AEI/10.13039/501100011033a), Marie Sklodowska-Curie (grant agreement No 702057 [CLIMIFUN]), and a project PAIDI 2020 from the Junta de Andalucía (P20_00879) |
| Fernando T. Maestre | European Research Council (ERC Grant agreement 647038 [BIODESERT]), Generalitat Valenciana (CIDEGENT/2018/041) |
| Maarja Öpik | European Regional Development Fund (Centre of Excellence EcolChange; TK131) |
| Mari Moora | Estonian Research Council, PRG1065 |
| Martin Zobel | European Regional Development Fund (Centre of Excellence EcolChange), Estonian Research Council (PRG1065). |
| Mikk Espenberg | Estonian Research Council, PRG352; European Regional Development Fund (Centre of Excellence EcolChange; TK131) |
| Annemieke Verbeken | Funding BOF (bijzonder onderzoeksfonds) Ghent University |
| Casper Nyamukondiwa | Botswana International University of Science and Technology |
| César Marín | Fondecyt project No. 1190642 (ANID – Chile) |
| Darta Klavina | ERDF project No. 1.1.1.2/VIAA/2/18/298 |
| Eduardo Nouhra | Secretaria de Ciencia y Técnica (SECYT) of Universidad Nacional de Córdoba and CONICET |
| Elisabeth Machteld Biersma | NERC‐CONICYT Grant NE/P003079/1 and Carlsberg Foundation Grant CF18‐0267 |
| Eske De Crop | Funding BOF (bijzonder onderzoeksfonds) Ghent University |
| Gregory Bonito | US National Science Foundation DEB 1737898 |
| Inga Hiiesalu | Estonian Research Council grant PSG1170 |
| Jelena Ankuda | The research work carried out to obtain the results has been funded by the EEA Financial Mechanism Baltic Research Programme in Estonia. A number of the project is: “EMP442”. |
| József Geml | Funding: Lendület Programme (No. 96049) of the Hungarian Academy of Sciences and the Eötvös Loránd Research Network |
| Juha M. Alatalo | Funding: Qatar Petroleum, QUEX-CAS-QP-RD-18/19 |
| Kadri Põldmaa | Estonian Research Council, PRG1170 |
| Kari Anne Bråthen | EEA Financial Mechanism Baltic Research Programme (EMP442) |
| Kęstutis Armolaitis | The research work carried out to obtain the results has been funded by the EEA Financial Mechanism Baltic Research Programme in Estonia. A number of the project is: “EMP442”. |
| Kevin K. Newsham | NERC core funding to the BAS Biodiversity, Evolution and Adaptation Team |
| Kristel Panksep | Estonian Science Foundation (P190250PKKH) |
| Linda Hansson | IRIS scholarship for progressive and ambitious women |
| Malka Saba | Higher Education Commision (HEC), Islamabad, Pakistan for indigenous and International research support initiative program (IRSIP) scholarships |
| Maria Tuomi | EEA Financial Mechanism Baltic Research Programme (EMP442) |
| Meike Piepenbring | Volkswagen Foundation |
| Peter E. Mortimer | "High-End Foreign Experts" in the High-Level Talent Recruitment Plan of Yunnan Province , 2021 |
| Rein Drenkhan | The Estonian Research Council grants PSG136 and PRG1615. |
| Roberto Garibay-Orijel | CONACYT-Ciencia Básica Project 2392669 |
| Roberto Godoy | Fondecyt project No. 1190642 (ANID – Chile) |
| Sunil Mundra | Start-up grant from research council of UAE University (#G00003320) |
| Terry W. Henkel | U.S. National Science Foundation grants DEB-0918591 and DEB-1556338 |
| Tomas Roslin | European Research Council (Synergy Grant 856506 – LIFEPLAN); Academy of Finland grant 322266 |
| Vladimir E. Fedosov | Russian Ministry of Science and Higher Education (# 075-15-2021-1396) |
| Vladimir G. Onipchenko | Russian Ministry of Science and Higher Education (# 075-15-2021-1396) |
| Alexandre Antonelli | Swedish Research Council (2019-05191), the Swedish Foundation for Strategic Research (FFL15-0196) and the Royal Botanic Gardens, Kew. |
